# Loss of Single‐Domain Function in a Modular Assembly Line Alters the Size and Shape of a Complex Polyketide

**DOI:** 10.1002/anie.201911315

**Published:** 2019-10-30

**Authors:** Huiyun Peng, Keishi Ishida, Christian Hertweck

**Affiliations:** ^1^ Department of Biomolecular Chemistry Leibniz Institute for Natural Product Research and Infection Biology (HKI) Beutenbergstrasse 11a 07745 Jena Germany; ^2^ Faculty of Biological Sciences Chair for Natural Product Chemistry Friedrich Schiller University Jena 07743 Jena Germany

**Keywords:** biosynthesis, module skipping, natural products, polyketide synthases, synthetic biology

## Abstract

The structural wealth of complex polyketide metabolites produced by bacteria results from intricate, highly evolved biosynthetic programs of modular assembly lines, in which the number of modules defines the size of the backbone, and the domain composition controls the degree of functionalization. We report a remarkable case where polyketide chain length and scaffold depend on the function of a single β‐keto processing domain: A ketoreductase domain represents a switch between diverging biosynthetic pathways leading either to the antifungal aureothin or to the nematicidal luteoreticulin. By a combination of heterologous expression, mutagenesis, metabolite analyses, and in vitro biotransformation we elucidate the factors governing non‐colinear polyketide assembly involving module skipping and demonstrate that a simple point mutation in type I polyketide synthase (PKS) can have a dramatic effect on the metabolic profile. This finding sheds new light on possible evolutionary scenarios and may inspire future synthetic biology approaches.

## Graphical abstract

**Biosynthetic switch**: Genetic and chemical analyses revealed a remarkable case where polyketide chain length and scaffold depend on the function of a single β‐keto processing domain of a multimodular polyketide synthase. A ketoreductase domain functions as a molecular switch between diverging pathways, leading either to the antifungal aureothin or to the nematicidal luteoreticulin.

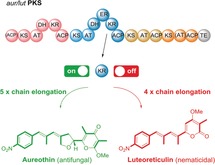

Modular type I polyketide synthases (PKSs) assemble a broad range of ecologically and pharmaceutically relevant molecules.^1^ In contrast to the chemical synthesis of complex polyketides, which often requires challenging, multistep transformations,^2^ type I PKSs connect and process simple acyl and malonyl thioester building blocks in a fully programmed fashion.^1^ Structural diversity results from variations in the biosynthetic program. Typically, an activated starter unit is loaded onto the PKS and propagated by modules that minimally consist of a ketosynthase (KS) domain catalyzing a Claisen condensation for C−C bond formation, an acyltransferase (AT) domain selecting and loading the malonyl extender unit, and an acyl carrier protein (ACP) domain serving as an anchor for the growing acyl chain. The polyketide chain is passed from one module to another until it is released from the PKS, usually catalyzed by a thioesterase (TE) domain.^3^ In each module optional ketoreductase (KR), dehydrogenase (DH), and enoylreductase (ER) domains determine to which degree the β‐keto groups of the intermediates are processed. Owing to the unidirectional propagation of the nascent chain bound to the assembly lines, the structures of the polyketide backbones and the corresponding modular PKSs are typically colinear.^1^ Thus, it is generally feasible to predict the basic polyketide structures based on the number and architectures of the PKS modules, and vice versa. (Figure 1) This co‐linearity rule has been successfully applied for genome mining^4^ and rational biosynthetic engineering approaches.^5^ Furthermore, it provides a model for the evolution of polyketide diversity; whereas gene duplications or deletions would lead to different chain lengths (number of modules), the gain or loss of encoded domain functions would influence the degree of β‐keto processing (composition of modules).^6^

**Figure 1:**
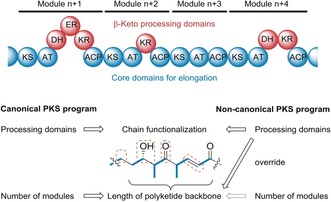
Co‐linearity rule in modular type I PKSs. MCoA: malonyl CoA, mMCoA: methylmalonyl‐CoA, KS: ketosynthase, AT: acyltransferase, DH: dehydratase, ER: enoylreductase, KR: ketoreductase, ACP: acyl carrier protein, TE: thioesterase.

To gain insight into such evolutionary processes and to emulate possible recombination scenarios we have studied in detail the biosynthesis of structurally related bacterial polyketides that share characteristic nitroaryl and pyrone moieties.^7^ Functional analyses of the assembly lines for the prototype of this family, the antifungal and antiproliferative agent aureothin (*aur*, **1**)^8^ and a higher homologue, neoaureothin (*syn*. spectinabilin),^9^ revealed that the involved PKSs breach with the colinearity rule.^10^ Specifically, the first modules and the penultimate AT domains are used iteratively.^11^ Based on the deduced biosynthetic program of the *aur* PKS we have engineered an artificial pathway for the nematicide luteoreticulin^12^ (*syn*. griseulin,^13^ **2**).^14^ This congener has a reduced chain length compared to **1**, an isomeric pyrone ring, and an altered substitution pattern. Although it has been feasible to morph the *aur* PKS into an artificial luteoreticulin (a*lut*) assembly line (Supporting Information, Figure S1),^14^ in the plethora of sequenced bacterial genomes a genuine gene cluster coding for luteoreticulin biosynthesis has not yet been detected. Here we elucidate the true biosynthetic origin of luteoreticulin and show the unexpected impact of single loss‐of‐function mutations of a modular PKS on the metabolite scaffold.

Prompted by the surprising observation that the aureothin producer strain *Streptomyces thioluteus* produces minute amounts of **2**, we revisited reported luteoreticulin producers (*S. luteoreticuli* 12 and *S. griseus* 15) and noted that **1** was also detected in their fermentation broths. To test the possibility that **2** is a side product of the *aur* pathway we investigated a heterologous host exclusively expressing the *aur* biosynthesis gene cluster (*S. albus*::pHJ48).7a By metabolic profiling of an up‐scaled culture of this designated producer strain we detected small amounts of **2**. Given that both polyketide metabolites (**1** and **2**) differ in both size and shape, it is remarkable that they seem to derive from the same modular assembly line.

The formation of **2** as a byproduct of the *aur* PKS (Figure 2 B) could be rationalized by erratic substrate processing or by non‐functional catalytic domains. A retro‐biosynthetic analysis suggested that the pyrone ring would result from the cyclization of an enol intermediate. The requisite carbonyl group would require an impaired β‐keto reduction after three rounds of elongations. Thus, we postulated that the ketoreductase domain (KR2) in module 2 (AurB) constitutes a branching point for the *lut* and *aur* pathways.

**Figure 2:**
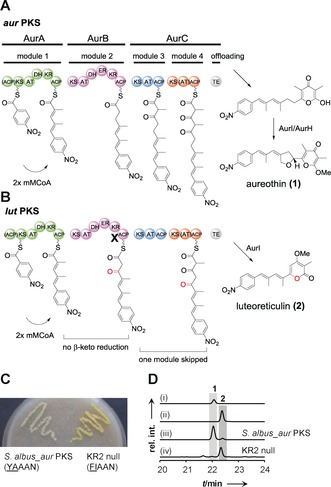
Dysfunctional KR domain in the *aur* biosynthetic pathway leads to the production of **2**. A) Non‐colinear polyketide biosynthesis in the *aur* pathway. Inactive ACP0 and AT4 are indicated with brackets. B) Model for *lut* biosynthesis. β‐Keto reduction does not take place in AurB, one module in AurC is skipped. C) *S. albus*::pHY127 (KR2 null mutant) shows strong yellow pigmentation on MS agar plate. The catalytic residue tyrosine was mutated to phenylalanine in YAAAN motif to create KR2 null mutant. D) HPLC profiles of (i) reference of **1**, (ii) reference of **2**, (iii) metabolites of heterologous expression strain *S. albus*::pHJ48 (*S. albus_aur* PKS), and (iv) KR2 null mutant, UV detection is at 350 nm.

To probe this hypothesis we scrutinized the KR2 domain. Alignment with other known KR domains (Figure S2) indicated that KR2 belongs to B1‐type KRs, which are characterized by a diagnostic LDD motif (VDD in KR2) and the absence of a Trp eight residues upstream of the catalytic Tyr.3a We also identified the catalytic Lys, Tyr, and Ser moieties that aid in binding and reducing polyketide intermediates. The only deviation is that leucine is replaced by Val in the conserved LDD motif. However, this replacement occasionally appears in other active KR domains^16^ (e.g., Nys3 and Nys12, Figure S2) and is thus unlikely to influence the reductive activity. Since KR2 is obviously functional, an impaired β‐keto reduction could result from a limited supply of the essential cofactor NADPH or from a slow turnover rate.

To test whether a complete shutdown of KR2 has an impact on the production of **2**, we inactivated the KR2 domain. Therefore, we replaced tyrosine with phenylalanine in the catalytic motif YAAAN by site‐directed mutagenesis of the PKS gene cloned into plasmid pHJ48 (Figures 2 B and S3). The resulting mutated plasmid (pHY127) was introduced into expression host *S. albus* by conjugation to generate the KR2 null mutant (*S. albus*::pHY127).

The metabolic profiles of *S. albus*::pHY127 and *S. albus*::pHJ48 were compared. A strong yellow pigmentation indicated an altered metabolite spectrum of the KR2 null mutant (Figure 2 C). HPLC‐MS analyses of the culture extracts showed that the production of **2** increased dramatically in the KR2 null mutant (50.8 mg L^−1^), whereas **1** could only be detected in trace amounts (0.36 mg L^−1^) (Figure 2 D, trace iv and Figure S7). Notably, production of **2** in the KR2 null mutant was 500‐fold higher than in the strain with the artificial *lut* (a*lut*) PKS (0.1 mg L^−1^).^14^ This finding strongly suggests that luteoreticulin production could result of a single loss of function in AurB. As a consequence of the impaired ketoreduction in module 2 the polyketide intermediate obviously skips a downstream module.

To gain insight into the impact of incomplete β‐keto processing we generated two additional mutants, one that is deficient in dehydration, and another one that lacks a functional ER domain. Therefore, we replaced the catalytic His of the HVVLGSTLVP motif3a of DH2 by Phe, yielding DH2 null mutant *S. albus*::pHY140 (Figure S4). To obtain the ER2 null mutant, *S. albus*::pHY147 (Figure S5), we changed the conserved NADPH‐binding motif GGVGMA3a to SPVGMA in ER2. HPLC analysis of the metabolic profile of the DH2 null mutant showed that in lieu of **1** and **2**, which can only be detected in trace amounts, several new compounds (**3**–**5**) are produced (Figure 3 B, trace iii). These metabolites were isolated in pure form by preparative HPLC to obtain **3** (6.4 mg), **4** (24.7 mg), and **5** (3.6 mg) from 1 L culture of DH2 null mutant (*S. albus*::pHY140), and their structures were elucidated by 1D and 2D NMR analysis (Figure 3 A, Figures S8–S28, and Tables S1–S3). The absolute configurations of **3** and **4** were determined by the combination of in silico analyses^17^ and chemical derivatization (Figures S29–S43, Tables S4–S6).

**Figure 3:**
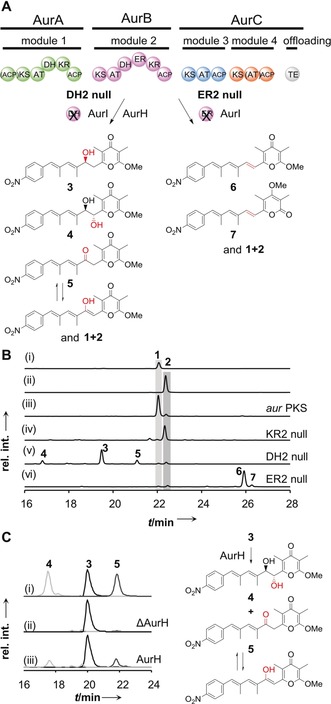
Impact of dysfunctional DH and ER domains on polyketide size and shape. A) Module skipping does not occur in DH2 null and ER2 null strains. Traces of **2** formed in all mutants due incomplete ketoreduction in AurB. B) HPLC profiles of (i) aureothin reference **1**, (ii) luteoreticulin reference **2**, (iii) metabolites of *S. albus*::pHJ48 (*aur*PKS), (iv) pHY127 (KR2 null mutant), (v) pHY140 (DH2 null mutant), (vi) pHY147 (ER2 null mutant). UV detection is at 350 nm. C) Recombinant AurH converts compound **3** to **4** and **5** in vitro. Extracted ion chromatogram of (i) references of **3**, **4**, and **5**, (ii) control experiment with heat‐inactivated AurH, (iii) biotransformation of **3** with AurH.

In agreement with the domain set of the mutated module 2 (DH2 null), compound **3** is a congener of **1** with a hydroxyl group at C8 that results from incomplete β‐keto processing. Compound **4** has an additional hydroxyl group at C7, which is likely introduced by the cytochrome P450 monooxygenase AurH.^18^ Compound **5** differs from **3** in a keto group at C8. This keto group might result from a) impaired ketoreduction, or b) oxidation of **3**, possibly by AurH. To elucidate the biogenesis of these oxygenated compounds, we heterologously produced AurH in *E. coli* used recombinant AurH for biotransformation experiments. In vitro assays showed that AurH transforms **3** into **4** and **5** (Figures 3 C and S58). Consequently, **4** results from AurH‐mediated C7‐hydroxylation, and **5** is formed by the oxidative route,^19^ which is plausible since an impaired KR at this stage would channel the intermediate into the *lut* pathway.

From the HPLC profile of the ER2 null mutant, we detected two new compounds, **6** and **7** (Figure 3 B, trace vi). The structures of **6** and **7** were determined by 1D and 2D NMR analyses of the isolated and purified compounds (Figures 3 A and S44–S57, and Tables S7 and S8). Both **6** and **7** feature double bonds at the respective C7‐C8 positions that result from ketoreduction and dehydratation, and differ only in the configuration of the *O*‐methylated pyrone ring (α‐ or γ‐position), which is mediated by the methyltransferase AurI.^20^ It is remarkable that trace amounts of **2** are detectable in all mutants. The formation of **2** can be explained by the non‐quantitative reduction of the β‐keto group in module 2 as observed in the wild‐type PKS (*S. albus*::pHJ48). Traces of **1** in the DH2 null mutant can be explained by non‐enzymatic dehydration of the hydroxy intermediate, or by the involvement of a long‐range acting DH domain as in iso‐migrastatin biosynthesis.^21^

The key message of these mutational experiments is, however, that the biosynthesis of **2** primarily depends on the dysfunction of the KR. Furthermore, it is remarkable that no module skipping takes place when the β‐keto group is processed into either β‐hydroxyl or enoyl groups. This finding indicates that the unreduced β‐keto group in module 2 is essential for the skipping process required for *lut* biosynthesis. Since AT4 is smaller than typical AT domains, and the function of AT4 can be substituted by AT3 from the penultimate module,^14^ module 4 is likely skipped.

To test this hypothesis, we replaced AT4 with AT3 in the KR2 null mutant. HPLC‐MS analysis revealed that **2** was still produced as the main product of the KR2 null+aAT4 mutant (*S. albus*::pHY145) (Figures 4 A–C and S6). Consequently, the AT domain exchange does not affect the skipping of the fourth module (Figure 4 C and D). A plausible explanation for this observation is that the KS4 domain solely plays a role as a gatekeeper that recognizes the structure of the polyketide chain. The required β,δ‐diketo thioester intermediate for pyrone formation could be generated by the iterative use of module 3 (*aur* pathway) or by the sequential single elongation of mutated module 2 and module 3 (*lut* pathway). While substrate specificities of KS domains are best studied for *trans*‐AT PKSs,^22^ it is astonishing that the *aur* KS2 domain downstream of iterative module 1 exhibits a similar gatekeeping function.^23^

**Figure 4:**
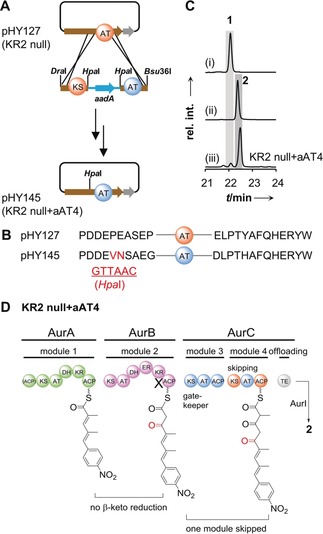
Gatekeeping of KS domain controls module skipping in luteoreticulin biosynthesis. A) Workflow to generate the aAT4 mutant (*S. albus*::pHY145). B) Amino acid sequences of original and mutated sites (introduced by restriction enzyme *Hpa*I). C) HPLC profiles of (i) aureothin reference (**1**), (ii) luteoreticulin reference (**2**), and (iii) metaboilites of KR2 null+aAT4 mutant. UV detection is at 350 nm. D) Module skipping is not influenced by AT4 activity.

Seminal studies have shown that mutagenesis and domain swaps of modular PKS lead to mainly predictable derivatives of the parent polyketide backbones.5a, 24 These genetic manipulations have led to a broad range of compounds with diverse substitution patterns, yet the size and overall scaffold of the polyketides has remained unaffected. We report a radically different scenario where a single loss of domain function or even a single point mutation of a modular PKS leads to a product with altered size and shape. This is an unusual mechanism by which structural diversity of biologically active polyketide products is created and illustrates an overlooked ability of PKS to breach with the colinearity rule on the basis of the intermediates’ redox state. Together with recently reports on keto‐processing PKS domains that are toggled on and off in an iterative module^25^ or have become dysfunctional during evolution^26^ to create structural diversity, these insights may guide future approaches to generate polyketide diversity by PKS engineering.

## Conflict of interest

The authors declare no conflict of interest.

## Supporting information

As a service to our authors and readers, this journal provides supporting information supplied by the authors. Such materials are peer reviewed and may be re‐organized for online delivery, but are not copy‐edited or typeset. Technical support issues arising from supporting information (other than missing files) should be addressed to the authors.

Supplementary
