# Supplementary material for: Loss of Single‐Domain Function in a Modular Assembly Line Alters the Size and Shape of a Complex Polyketide
Source: Angew Chem Int Ed Engl. 2019 Oct 30;58(50):18252–6. doi: 10.1002/anie.201911315 (PMC6916388; doi:10.1002/anie.201911315)
Supplement: Supplementary file 1 — Supplementary [file ANIE-58-18252-s001.pdf]

Angewandte Chemie
Eine Zeitschrift der Gesellschaft Deutscher Chemiker

Supporting Information

Loss of Single-Domain Function in a Modular Assembly Line Alters the Size and Shape of a Complex Polyketide

*Huiyun Peng, Keishi Ishida, and Christian Hertweck**

anie_201911315_sm_miscellaneous_information.pdf

SUPPLEMENTARY METHODS

Strains and media
General DNA procedures
Construction of KR2 null heterologous expression plasmid
Construction of DH2 null heterologous expression plasmid
Construction of ER2 null heterologous expression plasmid
Construction of KR2 null and AT4 exchange heterologous expression plasmid
General analytical procedures
Reversed-phase HPLC analysis and metabolite quantification of the EtOAc extracts from *S. albus* mutant strains
LC-MS/MS analysis for identification of small amount of aureothin from the EtOAc extract of *S. albus*::pHY127
Isolation of aureothin congeners from *S. albus*::pHY140
Isolation of aureothin congeners from *S. albus*::pHY147
Overproduction of AurH and protein purification
In vitro reconstitution of AurH activity
Preparation of (*S*)- and (*R*)-MTPA esters of **3**
Preparation of acetonide of **4**

SUPPLIMENTARY TABLES

Table S1. ^{1}H and ^{13}C NMR data for 8-hydroxy-7-deoxyaureothin (**3**) in $CDCl_3$ at 300K.
Table S2. ^{1}H and ^{13}C NMR data for 7,8-dihydroxy-7-deoxyaureothin (**4**) in CD_3OD at 300K.
Table S3. ^{1}H and ^{13}C NMR data for 8-oxo-7-deoxyaureothin (**5**) in $CDCl_3$ at 300K.
Table S4. ^{1}H and ^{13}C NMR data for (*S*)-MTPA ester of **3** in $CDCl_3$ at 300K.
Table S5. ^{1}H and ^{13}C NMR data for (*R*)-MTPA ester of **3** in $CDCl_3$ at 300K.
Table S6. ^{1}H and ^{13}C NMR data for acetonide of **4** in $CDCl_3$ at 300K.
Table S7. ^{1}H and ^{13}C NMR data for 7-dehydro-7-deoxyaureothin (**6**) in $CDCl_3$ at 300K.
Table S8. ^{1}H and ^{13}C NMR data for 2-pyrone-7-dehydro-7-deoxyaureothin (**7**) in $CDCl_3$ at 300K.

SUPPLEMENTARY FIGURES

Figure S1. The artificial luteoreticulin PKS.
Figure S2. Sequence alignment of KR domains.
Figure S3. Schematic strategy to generate KR2 inactive mutant.
Figure S4. Schematic strategy to generate DH2 inactive mutant.
Figure S5. Schematic strategy to generate ER2 inactive mutant.
Figure S6. Schematic strategy to generate KR2 inactive and AT4 exchange mutant.
Figure S7. LC-MS/MS analysis of aureothin (**1**).
Figure S8. High-resolution MS spectrum of **3** in positive ion mode.
Figure S9. Selected ^{1}H-^{1}H COSY and HMBC correlations of **3**.
Figure S10. ^{1}H NMR spectrum of 8-hydroxy-7-deoxyaureothin (**3**) in $CDCl_3$ at 300 K.
Figure S11. ^{13}C NMR spectrum of 8-hydroxy-7-deoxyaureothin (**3)** in $CDCl_3$ at 300 K.
Figure S12. ^{1}H-^{1}H COSY spectrum of 8-hydroxy-7-deoxyaureothin (**3**) in $CDCl_3$ at 300 K.
Figure S13. HSQC spectrum of 8-hydroxy-7-deoxyaureothin (**3**) in $CDCl_3$ at 300 K.
Figure S14. HMBC spectrum of 8-hydroxy-7-deoxyaureothin (**3**) in $CDCl_3$ at 300 K.
Figure S15. High-resolution MS spectrum of **4** in positive ion mode.
Figure S16. Selected ^{1}H-^{1}H COSY and HMBC correlations of **4**.
Figure S17. ^{1}H NMR spectrum of 7,8-hydroxy-7-deoxyaureothin (**4**) in CD_3OD at 300 K.
Figure S18. ^{13}C NMR spectrum of 7,8-hydroxy-7-deoxyaureothin (**4**) in CD_3OD at 300 K.
Figure S19. ^{1}H-^{1}H COSY spectrum of 7,8-hydroxy-7-deoxyaureothin (**4**) in CD_3OD at 300 K.
Figure S20. HSQC spectrum of 7,8-hydroxy-7-deoxyaureothin (**4**) in CD_3OD at 300 K.

Figure S21. HMBC spectrum of 7,8-hydroxy-7-deoxyaureothin (**4**) in CD_3OD at 300 K.
Figure S22. High-resolution MS spectrum of **5** in positive ion mode.
Figure S23. Selected ^1H-^1H COSY and HMBC correlations of **5**.
Figure S24. ^1H NMR spectrum of 8-oxo-7-deoxyaureothin (**5**) in $CDCl_3$ at 300 K.
Figure S25. ^{13}C NMR spectrum of 8-oxo-7-deoxyaureothin (**5**) in $CDCl_3$ at 300 K.
Figure S26. ^1H-^1H COSY spectrum of 8-oxo-7-deoxyaureothin (**5**) in $CDCl_3$ at 300 K.
Figure S27. HSQC spectrum of 8-oxo-7-deoxyaureothin (**5**) in $CDCl_3$ at 300 K.
Figure S28. HMBC spectrum of 8-oxo-7-deoxyaureothin (**5**) in $CDCl_3$ at 300 K.
Figure S29. Determination of the absolute configuration of **3** and **5**.
Figure S30. High-resolution MS spectrum (*S*)-MTPA ester of **3** in positive ion mode.
Figure S31. ^1H NMR spectrum of (*S*)-MTPA ester of **3** in $CDCl_3$ at 300 K.
Figure S32. HSQC spectrum of (*S*)-MTPA ester of **3** in $CDCl_3$ at 300 K.
Figure S33. HMBC spectrum of (*S*)-MTPA ester of **3** in $CDCl_3$ at 300 K.
Figure S34. High-resolution MS spectrum (*R*)-MTPA ester of **3** in positive ion mode.
Figure S35. ^1H NMR spectrum of (*R*)-MTPA ester of **3** in $CDCl_3$ at 300 K.
Figure S36. HSQC spectrum of (*R*)-MTPA ester of **3** in $CDCl_3$ at 300 K.
Figure S37. HMBC spectrum of (*R*)-MTPA ester of **3** in $CDCl_3$ at 300 K.
Figure S38. High-resolution MS spectrum of acetonide of **3** in positive ion mode.
Figure S39. ^1H NMR spectrum of acetonide of **4** in $CDCl_3$ at 300 K.
Figure S40. ^{13}C NMR spectrum of acetonide of **4** in $CDCl_3$ at 300 K.
Figure S41. HSQC spectrum of acetonide of **4** in $CDCl_3$ at 300 K.
Figure S42. HMBC spectrum of acetonide of **4** in $CDCl_3$ at 300 K.
Figure S43. NOESY spectrum of acetonide of **4** in $CDCl_3$ at 300 K.
Figure S44. High-resolution MS spectrum of **6** in positive ion mode.
Figure S45. Selected HMBC and ROESY correlations of **6**.
Figure S46. ^1H NMR spectrum of 7-dehydro-7-deoxyaureothin (**6**) in $CDCl_3$ at 300 K.
Figure S47. ^{13}C NMR spectrum of 7-dehydro-7-deoxyaureothin (**6**) in $CDCl_3$ at 300 K.
Figure S48. HSQC spectrum of 7-dehydro-7-deoxyaureothin (**6**) in $CDCl_3$ at 300 K.
Figure S49. HMBC spectrum of 7-dehydro-7-deoxyaureothin (**6**) in $CDCl_3$ at 300 K.
Figure S50. ROESY spectrum of 7-dehydro-7-deoxyaureothin (**6**) in $CDCl_3$ at 300 K.
Figure S51. High-resolution MS spectrum of **7** in positive ion mode.
Figure S52. Selected HMBC and ROESY correlations of **7**.
Figure S53. ^1H NMR spectrum of 2-pyrone-7-dehydro-7-deoxyaureothin (**7**) in $CDCl_3$ at 300 K.
Figure S54. ^{13}C NMR spectrum of 2-pyrone-7-dehydro-7-deoxyaureothin (**7**) in $CDCl_3$ at 300 K.
Figure S55. HSQC spectrum of 2-pyrone-7-dehydro-7-deoxyaureothin (**7**) in $CDCl_3$ at 300 K.
Figure S56. HMBC spectrum of 2-pyrone-7-dehydro-7-deoxyaureothin (**7**) in $CDCl_3$ at 300 K.
Figure S57. ROESY spectrum of 2-pyrone-7-dehydro-7-deoxyaureothin (**7**) in $CDCl_3$ at 300 K.
Figure S58. SDS-PAGE of AurH purification.

SUPPLEMENTARY REFERENCES

SUPPLEMENTARY METHODS

Strains and media

E. coli strain TOP10 (Invitrogen) and XL1-Blue (Agilent) are served for routine subcloning. *E. coli* HB101/pRK2013 and BW25113/pIJ790 were employed for triparental conjugation[1] and PCR targeting procedures,[2] respectively. *E. coli* strains were cultured in Luria-Bertani (LB) medium supplemented with spectinomycin (100 μg mL^{-1}, Carl Roth), apramycin (30 μg mL^{-1}, Sigma-Aldrich), or chloramphenicol (25 μg mL^{-1}, Carl Roth) for plasmid selection. *S. albus*, was used as host strain for heterologous expression experiment, was kindly provided by Prof. Jose A. Salas (University of Oviedo, Spain). For metabolites production, *Streptomyces* strains were grown with orbital shaking at 30 °C in TSB (Tryptic Soy Broth) for 1 day. This seed culture (10 mL) was used to inoculate 100 mL M10[3] medium. After 5−6 days cultivation with orbital shaking at 30 °C, fermentation was stopped. For sporulation, *Streptomyces* strains were grown on MS (mannitol soya flour)[4] agar plates. Conjugation was performed on MS agar supplemented with $MgCl_2$ (10 mM) and overlaid next day with nalidixic acid (20 μg mL^{-1}, Carl Roth) and apramycin (30 μg mL^{-1}, Sigma-Aldrich).

General DNA procedures

DNA isolation, plasmid preparation, restriction digests, PCR, gel electrophoresis, ligation reactions and transformation were performed according to standard methods[5] for *E. coli*. Restriction enzyme digestions and PCR amplified DNA fragments were purified from agarose gel by Monarch DNA Gel Extraction Kit (New England Biolabs).

Construction of KR2 null heterologous expression plasmid

An *E. coli-Streptomyces* shuttle plasmid (pHJ48)[6] including the whole aureothin biosynthetic gene cluster was digested with *Kpn*I. The 15 kb DNA fragment was inserted into pCR-Blunt vector (Invitrogen) to yield pHY115, which was used for further gene cloning strategies. The PCR-targeting approach was used to insert the spectinomycin resistance gene cassette into the point-mutation site. The spectinomycin resistance gene cassette was amplified from pIJ778[4] with primer pair Spec-KR2-fw (5'-GTC GCG GGC ACC CTC GGC TCG GCC GCC CAG GCC AAC ttt aaa ATT CCG GGG ATC CGT CGA CC-3') and -rv (5'-GTG GGC GGC CAG GGC GTC GAG GAA GGC GTT GGC GGC aat att TGT AGG CTG GAG CTG CTT C-3'), inducing the point mutation sequence using the primers. The 1.5 kb spectinomycin resistance gene cassette was introduced into *E. coli* BW25113/pIJ790/pHY115. Plasmid pHY118 was then generated with a spectinomycin resistance gene cassette inserted into pHY115 *via* recombination. The 16 kb pHY118/*Kpn*I fragment was then inserted into pHJ48/*Kpn*I. The resulting plasmid pHY123 was digested by *Dra*I and *Ssp*I, and the 35 kb DNA fragment was further re-ligated to produce the point mutation site (YAAAN to FIAAN). To facilitate the introduction of this plasmid into the *Streptomyces* host by conjugation, *oriT* was inserted at *Xba*I site *via* NEBuilder HiFi DNA Assembly Cloning Kit (New England Biolabs), generating plasmid pHY127.

Construction of DH2 null heterologous expression plasmid

The restriction site *Hpa*I was induced into pHJ48-oriT to generate pHY130. Then the DNA fragment coding for the DH2 domain was excised from pHY130 by *Fsp*AI and *Hpa*I. This fragment was inserted into pCR-Blunt to generate pHY137. The site-directed mutagenesis was induced in pHY137 by primer pairs DH2-inactivation-fw (5'- GCC GAC TTC GTC GTG CTC GGC TCC ACG CTC-3') and DH2-inactivation-rv (5'- GCC GAG CAC GAC GAA GTC GGC CAG CCA GGA C-3'). The gene region for the mutated DH2 domain was amplified from the resulting plasmid pHY138 with primer pair iDH2-AS-fw (5'-GGC CCC CGA TGC GGA GGA GTC GGT TGA CGC GGA GCC CGC GTC C-3') and iDH2-AS-rv (5'-ACC TCG TGC CAG GCG AAC GGG ATG CGC ACC TGC GAC GTG TCG GAC-3'). This DNA

fragment was assembled with the 33 kb pHY130/*Fsp*AI+*Hpa*I fragment to generate pHY140. The conserved motif HVVLGSTLVP was mutated into FVVLGSTLVP.

Construction of ER2 null heterologous expression plasmid

The ER2 domain encoding DNA fragment pHY123/*Fsp*AI+*Ssp*I was inserted into pCR-Blunt to generate pHY136. The site-directed mutagenesis was induced in pHY136 by primer pair ER2-inactivation-fw (5'-GCG GCG AGC CCC GTC GGC ATG GCC GCC GTC-3') and ER2-inactivation-rv (5'-CAT GCC GAC GGG GCT CGC CGC CGC GTG CAC-3'). The 2.2 kb DNA fragment containing the mutated ER2 domain was amplified from the resulting plasmid pHY139 with primer pair iER2-AS-fw (5'- GAG GCG TCC GAC ACG TCG CAG GTG CGC ATC CCG TTC GCC TGG C-3') and iER2-AS-rv1 (5'-CGT CGA GGA AGG CGT TGG CGG CAG CGT AGT TGG CCT GGG CGG C-3'). This DNA fragment was assembled with the 33 kb pHY123/*Fsp*AI+*Ssp*I fragment to generate pHY141. The *ori*T was inserted into the *Xba*I site of pHY141 for conjugation. In the resulting plasmid pHY147, the encoded conserved motif GGVGMA was mutated to SPVGMA, and the inactive motif FIAAN in KR2 was activated into YAAAN.

Construction of KR2 inactive and AT4 exchange heterologous expression plasmid

The plasmid pHY127 was introduced into *E.coli* BW25113/pIJ790 by electroporation. A 4.5 kb DNA fragment covering region KS4-*aadA*-AT3-ACP4 is obtained by digesting pYU59[7] with *Dra*I and *Bsu*36I. This DNA fragment was introduced into BW25113/pIJ790/pHY127. pHY144 was then generated as the spectinomycin cassette was inserted into pHY127 *via* recombination. The spectinomycin cassette was removed by *Hpa*I to generate plasmid pHY145. pHY145 was introduced into *S. albus* to generate a KR2-inactive and AT4-active mutant, *S. albus*::pHY145 (KR2 null+aAT4).

General analytical procedures

NMR spectra were measured on a Bruker Avance 600 with cryo probe. Spectra were referenced to the residual solvent peaks at δ_H = 7.24 / δ_C = 77.0 ppm for $CDCl_3$ and δ_H = 3.30 / δ_C = 49.0 ppm for CD_3OD. UV spectrum was obtained on a Shimadzu UV-1800 spectrometer for spectroscopy. Analytical RP-HPLC was performed on a Shimadzu LC10AD with SPD-M10Avp DAD and preparative RP-HPLC was performed on a Shimadzu LC8A with SPD-M20A DAD. HPLC-HR ESIMS measurements were carried out on a Thermo Fisher Scientific Exactive Orbitrap with an electrospray ion source using a Betasil 100-3 C_{18} column (2.1 × 150 mm) and an elution gradient [solvent A: water containing 0.1% formic acid, solvent B: acetonitrile, gradient: 5% B for 1 min, 5% to 98% B in 15 min, 98% B for 3 min, flow rate: 0.2 mL min^{-1}].

Reversed-phase HPLC analysis and metanbolite quantification of the ethylacetate extracts from *S. albus* mutant strains

After 5−6 days fermentation, 1 volume ethyl acetate was added to the culture, and stirred at room temperature for overnight. Then, the organic phase was dried over sodium sulfate and concentrated to dryness under reduced pressure. The residue was dissolved in 1 mL methanol. The obtained extract (10 µL) was analysed by reversed phase column HPLC (Symmetry C18 5 µm, 4.6 × 150 mm, Waters) using a gradient program with solvent A (water containing 0.1% trifluoroacetic acid (TFA)) and solvent B (acetonitrile), 20% B for 5 min, to 99% B in 30 min, kept 99% B for 5 min. The flow rate is 1 mL min^{-1}. UV was detected at a wavelength of 350 nm. The aureothin and luteoreticulin quantities were calculated from area sizes compare to standards except for the ethylacetate extract from *S. albus*::pHY127. The aureothin quantity of the ethylacetate extract from *S. albus*::pHY127 was calculated from peak height compare to standards, because one overlapping peak was interfered an area size determination.

LC-MS/MS analysis for identification of small amount of aureothin from the ethylacetate extract of *S. albus*::pHY127

LC-MS/MS measurements were carried out by Bruker HCT Ultra ion trap mass spectrometry (BrukerDaltonics, Bremen, Germany) coupled with an Agilent Technologies 1100 series liquid chromatogram system (Agilent, Waldbronn, Germany). The ionization mode was electro-spray (ESI), polarity positive, mass range mode ultra-scan, and nitrogen was used as a drying and nebulizer gas. The following parameters were applied: nebulizer 70 psi, dry gas 12 L min^{-1}, dry temperature 365 °C, scan range *m/z* 300−2000, No-of precursor ions 2. 10 µL of samples were subjected to a reversed-phase HPLC column Zorbax Eclipse XDB-C8 (Agilent, 5 µm, 4.6 × 150 mm, flow rate 1mL min^{-1}) using a gradient system with solvent A (water containing 0.1% formic acid), solvent B (acetonitrile), 10% B for 10 min to 99% B in 25 min and kept 99% B for 4 min, to 10% B in 1 min.

Isolation of aureothin congeners from *S. albus*::pHY140

One liter culture of *Streptomyces albus*::pHY140 was extracted with 1 L ethyl acetate three times and organic layers were dried over anhydrous sodium sulfate. After the solvent was removed under the reduced pressure, the extract was subjected to silica gel column chromatography (3 × 10 cm, silica gel 60, 0.040–0.063 µm) using dichloromethane/methanol stepwise system. The luteoreticulin containing fraction was subjected to semi-preparative RP-HPLC (phenomenex fusion-RP C18, diameter 4 µm, pore size 80 Å, 10 × 250 mm, flow rate 6 mL min^{-1}) using a gradient system; solvent A (water containing 0.1% TFA), B (100% acetonitrile), 30% B in 10 min to 100% B in 35 min, to yield a crude luteoreticulin. This crude fraction was further subjected to semi-preparative RP-HPLC (Nucleodur HTec C18 diameter 5 µm, pore size 100Å, 10 × 250 mm, flow rate 4 mL min^{-1}) using a gradient system; solvent A (water containing 0.1% TFA), B (100% acetonitrile), 30% B in 10 min to 100% B in 35 min, to yield a luteoreticulin (**2**,0.9 mg). The three aureothin congeners 8-hydroxy-7-deoxyaureothin (**3**), 7,8-dihydroxy-7-deoxyaureothin (**4**), 8-oxo-7-deoxyaureothin (**5**) containing fraction was subjected to semi-preparative RP-HPLC (phenomenex fusion-RP C18 diameter 4 µm, pore size 80Å, 10 × 250 mm, flow rate 6 mL min^{-1}) using a gradient system; solvent A (water containing 0.1% TFA), B (100% acetonitrile), 30% B in 10 min to 100% B in 35 min, to yield crude **3**, **4**, and **5**, respectively. The crude **3**, **4**, and **5** were further subjected to semi-preparative RP-HPLC (Nucleodur HTec C18 diameter 5 µm, pore size 100Å, 10 × 250 mm, flow rate 4 mL min^{-1}) using a gradient system; solvent A (water containing 0.1% TFA), B (100% acetonitrile), 30% B in 10 min to 100% B in 35 min, to yield 8-hydroxy-7-deoxyaureothin (**3**, 6.4 mg), 7,8-dihydroxyaureothin (**4**, 24.7 mg), and 8-oxo-7-deoxyaureothin (**5**, 3.6 mg), respectively.

8-Hydroxy-7-deoxyaureothin (**3**): pale yellow amorphous solid; HRMS: $[M+H]^+$ = 400.1756 (calculated for $C_{22}H_{26}NO_6$ 400.1755); NMR data see **Table S1**.

7,8-Dihydroxy-7-deoxyaureothin (**4**): pale yellow amorphous solid; HRMS: $[M+H]^+$ = 416.1705 (calculated for $C_{22}H_{26}NO_7$ 416.1704); NMR data see **Table S2**.

8-Oxo-7-deoxyaureothin (**5**): pale yellow amorphous solid; HRMS: $[M+H]^+$ = 398.1602 (calculated for $C_{22}H_{24}NO_6$ 398.1598); NMR data see **Table S3**.

Isolation of aureothin congeners from *S. albus*::pHY147

One liter culture of *Streptomyces albus*::pHY147 was extracted with 1 L ethyl acetate. Obtaining emulsion phase was filtered through Celite 545. The water phase was further extracted with 1 L ethyl acetate twice and organic layers were dried over anhydrous sodium sulfate. After the solvent was removed under the reduced pressure, the extract was subjected to silica gel column chromatography (3 × 13 cm, silica gel 60, 0.040–0.063 µm,

Merck) using dichloromethane/methanol stepwise system. The α-pyrone compounds containing fraction was subjected to preparative reversed-phase HPLC (Nucleosil 100-5C18, diameter 7 µm, pore size 100 Å, 21.2 × 250 mm, flow rate 15 mL min^{-1}) using a gradient system; solvent A (40% aqueous acetonitrile containing 0.1% TFA), solvent B (100% acetonitrile), 1% B for 10 min to 100% B in 30 min and kept 10 min, to obtain crude **6** and crude **7** fractions, respectively. Crude **6** and **7** fractions were further subjected to semi-preparative reversed-phase HPLC (Nucleodur HTec C18, diameter 5 µm, pore size 100 Å, 10 × 250 mm, flow rate 6 mL min^{-1}) using a 70% B isocratic system; solvent A (H_2O containing 0.1% TFA), solvent B (83% acetonitrile) to yield **6** (36.4 mg) and **7** (10.6 mg), respectively.

7-Dehydro-7-deoxyaureothin (**6**): yellow amorphous solid; HRMS: $[M+H]^+$ = 382.1643 (calculated for $C_{22}H_{24}NO_5$ 382.1649); NMR data see **Table S4**.

2-Pyrone-7-dehydro-7-deoxy-aureothin (**7**): yellow amorphous solid; HRMS: $[M+H]^+$ = 382.1647 (calculated for $C_{22}H_{24}NO_5$ 382.1649); NMR data see **Table S5**.

Overproduction of AurH and protein purification

The 20 µL glycerol stock of *E. coli* BL21 (DE3) codon plus harboring pMR21,[3] coding for a MalE-AurH fusion protein, was cultured in 10 mL LB medium containing appropriate antibiotics at 37 °C for overnight. Three mL of pre-culture was added to 300 mL LB medium with antibiotics and cultivated at 37 °C for 2–3 h until the OD_{600} reached 0.8–1.0. This main culture was put on ice for 30 min to cool down. Then 0.5 mM IPTG and 1.0 mM 5-aminolevulinate were added. This culture was further incubated at 20 °C for overnight. The cells were harvested by centrifuge and stored at −20 °C.
Frozen cells from 100 mL cultured cells were suspended in 25 mM Tris HCl (pH 7.5) buffer with 100 mM NaCl and 1 mg mL^{-1} lysozyme (Carl Roth) and then incubated at 37 °C for 30 min. This solution was sonicated by Sonoplus MS73 (Bandelin, 30% power, 5 cycles for 2 min, repeated at 5 times) on an ice. After the centrifugation of destroyed cells at 10,000 × *g* for 20 min at 4 °C, the supernatant was filtered by Chromafil (0.45 µm, Macherey-Nagel) and subjected to an Amylose column (1 mL, NEB) and washed with 15 mL of 25 mM Tris HCl (pH 7.5) buffer with 100 mM NaCl. The amylose resin binding MalE-AurH fusion protein in a column was resuspended with 25 mM Tris HCl (pH 7.5) buffer with 100 mM NaCl and 50 µL of Factor Xa (NEB). This reaction mixture was incubated at room temperature with shaking for 16 h and eluted AurH protein from Amylose column. After further elution by 3 mL of additional 25 mM Tris HCl (pH 7.5) buffer with 100 mM NaCl, eluent was subjected to pAmino benzamidine agarose (1 mL) column and incubated at room temperature with shaking for 1 h. AurH protein was eluted from pAmino benzamidine agarose column with additional 5 mL of 25 mM Tris HCl (pH 7.5) buffer with 100 mM NaCl. A combined eluents was concentrated by Amicon Ultra centrifugal filter 30 kDa (Merck Millipore) to obtain 4.0 mg of AurH (Figure S31). To this AurH solution, glycerol was added as a final concentration of 10% and stored at −80 °C.

***In vitro* reconstitution of AurH activity**

The *in vitro* oxygenase activity assay was carried out in analogy to the reported procedure.[3] The 250 µL reaction mixture consisted of 25 µL of spinach ferredoxin (Sigma-Aldrich, 100 µM), 25 µL of spinach ferredoxin-NADP-oxidoreductase (Sigma-Aldrich, 1 units mL^{-1}), 2.5 µL of NADPH (100 mM), 2.5 µL of baker's yeast glucose-6-phosphate dehydrogenase (Sigma-Aldrich, 100 U mL^{-1}), 2.5 µL of glucose-6-phosphate (Sigma-Aldrich, 1 M), 25 µL of AurH (100 µM), 2.5 µL of substrates (20 mM), and 162.5 µL of Tris HCl pH 7.5 (100 mM). This mixture was incubated at 30 °C with shaking for 16 h and was extracted with ethylacetate (2 x 500 µL). After the organic layers were dried over anhydrous sodium sulfate, the solvent was removed under the reduced pressure. The extract was redissolved in 100 µL methanol, of which 10 µL were injected to HPLC-MS, Bruker HCT Ultra ion trap mass spectrometry

coupled with an Agilent Technologies 1100 series liquid chromatogram system as above-mentioned method.

Preparation of (*S*) and (*R*)-MTPA esters of 3

To 2.0 mg of 8-hydroxy-7-deoxyaureothin (**3**), dry dichloromethane (200 μL), dry pyridine (100 μL), and excess amounts of (*R*)-α-methoxy-α-trifluoromethylphenylacetyl chloride (MTPA Cl) were added. The reaction mixture was stirred under the argon at room temperature for 2 h. To the resulting mixture, 1 mL of water was added and extracted with 1 mL dichloromethane. After repeated dichloromethane extraction, the combined extracts were dried over anhydrous sodium sulfate, and the solvent was removed under the reduced pressure. The residue was subjected to the reversed-phase HPLC (Nucleodur HTec C18 diameter 5 μm, pore size 100 Å, 10 × 250 mm, flow-rate at 6 mL min^{-1}) using a gradient system; solvent A (40% aqueous acetonitrile containing 0.1% TFA), solvent B (100% acetonitrile), 10% B in 10 min to 100% B in 30 min to yield (*S*)-MTPA ester of **3** (2.8 mg, HRMS: $[M+H]^+$ = 616.2156 (calculated for $C_{32}F_3H_{33}NO_8$ 616.2153). (*R*)-MTPA ester of **3** (2.3 mg, HRMS: $[M+H]^+$ = 616.2155 (calculated for $C_{32}F_3H_{33}NO_8$ 616.2153) was obtained from (*S*)-MTPA Cl according to the above-mentioned procedure.

Preparation of acetonide of 5

After 7,8-dihydroxy-7-deoxyaureothin (**5**, 830 μg, 2.0 μmol) was dissolved in dry dichloromethane, 2,2-dimethoxypropane (2.5 μL, 20 μmol) and pyridinium *p*-toluenesulfonate (50 μg, 0.2 μmol) were added to the solution. The reaction mixture was stirred under the argon at room temperature overnight. The resulting mixture was treated with trimethylamine (1.4 μL, 10 μmol) and 1 mL of saturated aqueous sodium bicarbonate. This solution was extracted with 1 mL of dichloromethane. After repeated dichloromethane extraction twice, the solvent was dried over anhydrous sodium sulfate, and the solvent was removed under reduced pressure. The residue was subjected to reversed-phase HPLC (Nucleodur HTec C18, diameter 5 μm, pore size 100 Å, 10 × 250 mm, flow-rate at 6 mL min^{-1}) using a gradient system; solvent A (40% aqueous acetonitrile containing 0.1% TFA), solvent B (100% acetonitrile), 10% B in 10 min to 100% B in 30 min to yield acetonide of **5** (800 μg, HRMS: $[M+H]^+$ = 456.2003 (calculated for $C_{25}H_{30}NO_7$ 456.2017)).

SUPPLEMENTARY TABLES

Table S1. ^{1}H and ^{13}C NMR data for 8-hydroxy-7-deoxyaureothin (**3**) in $CDCl_3$.

Position	^{1}H (J_{Hz})	^{13}C (mult)	HMBC correlations (^{1}H to ^{13}C)
1			
2		162.3 (s)	
2a	3.94 (s)	55.3 (q)	2, 3
3		99.2 (s)	
3a	1.72 (s)	6.7 (q)	2, 3, 4
4		180.9 (s)	
5		119.8 (s)	
5a	1.90 (s)	10.1 (q)	4, 5, 6
6		155.8 (s)	
7	2.80 (dd 14.3, 5.2)	37.5 (t)	5, 6, 8, 9
	2.89 (dd 14.3, 8.4)		5, 6, 8, 9
8	4.46 (brdd 8.4, 5.1)	75.0 (d)	6, 7, 9, 9a, 10
9		145.7 (s)	
9a	1.92 (s)	14.1 (s)	8, 9, 10
10	6.05 (s)	129.5 (d)	8, 9, 9a, 11a, 12
11		138.8 (s)	
11a	1.95 (s)	19.0 (q)	10, 11, 12
12	6.33 (s)	127.8 (d)	10, 11, 11a, 13, 14, 15
13		144.5 (s)	
14,15	7.34 (d 8.4)	129.4 (d)	12, 13, 14, 15, 16, 17, 18
16,18	8.12 (d 8.4)	123.3 (d)	13, 16, 17, 18
17		145.7 (s)	

Table S2. ^{1}H and ^{13}C NMR data for 7,8-dihydroxyaureothin (**4**) in CD_3OD.

Position	^{1}H (J_{Hz})	^{13}C (mult)	HMBC correlations (^{1}H to ^{13}C)
1			
2		164.7 (s)	
2a	4.12 (s)	56.6 (q)	2, 3
3		100.4 (s)	
3a	1.83 (s)	7.1 (q)	2, 3, 4
4		183.1 (s)	
5		121.3 (s)	
5a	2.03 (s)	9.8 (q)	4, 5, 6
6		159.0 (s)	
7	4.83 (d 8.5)	70.4 (d)	2, 5, 6, 8, 9
8	4.37 (d 8.5)	79.4 (d)	6, 7, 9, 9a, 10
9		139.0 (s)	
9a	2.00 (brd)	13.8 (s)	8, 9, 10
10	6.17 (s)	134.0 (d)	8, 9, 9a, 11a, 12
11		140.4 (s)	
11a	2.06 (s)	19.4 (q)	10, 11, 12
12	6.52 (s)	129.1 (d)	10, 11, 11a, 13, 14, 15
13		146.1 (s)	
14,15	7.51 (d 8.4)	130.8 (d)	12, 13, 14, 15, 16, 17, 18
16,18	8.20 (d 8.4)	124.4 (d)	13, 16, 17, 18
17		147.3 (s)	

Table S3. ^{1}H and ^{13}C NMR data for 8-oxo-7-deoxyaureothin (**5**) in $CDCl_3$.

Position	^{1}H (J_{Hz})	^{13}C (mult)	HMBC correlations (^{1}H to ^{13}C)
1			
2		162.4 (s)	
2a	3.89 (s)	55.5 (q)	2
3		99.9 (s)	
3a	1.84 (s)	6.9 (q)	2, 3, 4
4		180.7 (s)	
5		120.7 (s)	
5a	1.93 (s)	10.3 (q)	4, 5, 6
6		152.1 (s)	
7	4.04 (s)	40.3 (t)	5, 6, 8
8		194.9 (s)	
9		136.6 (s)	
9a	2.08 (s)	13.5 (s)	8, 9, 10
10	7.12 (s)	143.5 (d)	8, 9, 9a, 11a, 12
11		137.4 (s)	
11a	2.14 (s)	18.7 (q)	10, 11, 12
12	6.68 (s)	133.0 (d)	10, 11, 11a, 13, 14, 15
13		143.0 (s)	
14,15	7.46 (d 8.4)	129.9 (d)	12, 14, 15, 16, 17, 18
16,18	8.22 (d 8.4)	123.7 (d)	13, 16, 17, 18
17		146.7 (s)	

Table S4. ^{1}H and ^{13}C NMR data for (*S*)-MTPA ester of **3** in $CDCl_3$.

Position	^{1}H (J_{Hz})	^{13}C (mult)*	HMBC correlations (^{1}H to ^{13}C)
1			
2		162.0 (s)	2a, 3a
2a	3.94 (s)	55.4 (q)	
3		99.8 (s)	3a
3a	1.84 (s)	6.8 (q)	
4		180.5 (s)	3a, 5a
5		120.6 (s)	5a
5a	1.86 (s)	9.9 (q)	
6		152.6 (s)	5a, 7, 8
7	2.89 (dd 14.5, 9.2)<br>3.12 (dd 14.5, 3.9)	34.6 (d)	8
8	5.77 (dd 8.8, 4.1)	78.1 (s)	7, 9a
9		133.1 (s)	7, 9a, 10
9a	1.96 (s)	13.8 (s)	8, 10
10	6.18 (s)	133.6 (d)	8, 9a, 11a, 12
11		137.2 (s)	11a, 12
11a	1.99 (s)	18.6 (q)	10, 12
12	6.40 (s)	129.1 (d)	10, 11a
13		143.8 (s)	15, 17
14,18	7.40 (d 8.6)	129.3 (d)	12, 14, 18
15,17	8.20 (d 8.6)	123.3 (d)	14, 15, 17, 18
16		145.8 (s)	14, 15, 17, 18
19		165.6 (s)	8
20		84.3 (s)	21, 23
21	3.46 (s)	55.1 (q)	
22		131.5 (s)	24, 26
23, 27	7.36 (d 7.3)	126.5 (d)	23, 25, 27
24, 26	7.31 (t 7.3)	128.2 (d)	24, 26
25	7.38 (m)	129.3 (d)	23, 27
26	C-F_3	n.d.	

*; ^{13}C Chemical shifts assignments were obtained from HSQC and HMBC correlations.
n.d.; not determined.

Table S5. ^{1}H and ^{13}C NMR data for (*R*)-MTPA ester of **3** in $CDCl_3$.

Position	^{1}H (J_{Hz})	^{13}C (mult)*	HMBC correlations (^{1}H to ^{13}C)
1			
2		166.2 (s)	2a, 3a
2a	3.91 (s)	55.4 (q)	
3		99.8 (s)	3a
3a	1.85 (s)	6.9 (q)	
4		180.3 (s)	3a, 5a
5		120.5 (s)	5a
5a	1.93 (s)	10.0 (q)	
6		152.8 (s)	5a, 7, 8
7	2.93 (dd 14.6, 3.7)<br>3.14 (dd 14.6, 9.4)	32.2 (d)	8
8	5.70 (dd 9.4, 3.7)	78.3 (s)	7, 9a, 10
9		133.1 (s)	7
9a	1.85 (s)	13.2 (s)	8, 10
10	6.10 (s)	133.2 (d)	8, 9a, 11a, 12
11		137.4 (s)	10, 11a, 12
11a	1.97 (s)	18.5 (q)	10, 12
12	6.36 (s)	128.9 (d)	10, 11a, 14, 15
13		143.8 (s)	12, 16, 18
14,15	7.39 (d 8.6)	129.3 (d)	12, 14, 15
16,18	8.19 (d 8.6)	123.3 (d)	16, 18
17		145.9 (s)	12, 14, 16, 18
19		165.7 (s)	8
20		84.4 (s)	21, 23
21	3.39 (s)	55.2 (q)	
22		131.4 (s)	24, 26
23, 27	7.33 (d 7.6)	126.8 (d)	23, 25, 27
24, 26	7.30 (t 7.6)	128.3 (d)	23, 24, 26, 27
25	7.37 (t 7.6)	129.3 (d)	23, 27
28	(CF_3)	n.d.	

*; ^{13}C Chemical shifts assignments were obtained from HSQC and HMBC correlations.
n.d.; not determined.

Table S6. ^{1}H and ^{13}C NMR data for acetonide of **4** in $CDCl_3$.

Position	^{1}H (J_{Hz})	^{13}C (mult)	HMBC correlations (^{1}H to ^{13}C)
1			
2		162.6 (s)	2a
2a	4.00 (s)	56.0 (q)	
3		100.2 (s)	3a
3a	1.85 (s)	7.1 (q)	3a
4		180.3 (s)	3a, 5a
5		120.7 (s)	5a, 7
5a	2.01 (s)	10.1 (q)	
6		153.2 (s)	5a, 7, 8
7	5.42 (d 7.5)	74.4 (d)	8
8	4.95 (d 7.5)	81.9 (d)	10
9		131.8 (s)	8, 9a, 10
9a	1.63 (s)	15.6 (s)	8, 10
10	6.23 (s)	130.1 (d)	8, 9a, 12
11		138.1 (s)	10, 11a
11a	1.89 (s)	18.9 (q)	10, 12
12	6.12 (s)	128.1 (d)	10, 11a, 14, 15
13		144.1 (s)	12, 16, 18
14,15	7.31 (d 8.7)	129.6 (d)	12, 14, 15
16,18	8.16 (d 8.7)	123.5 (d)	14, 15, 16, 18
17		146.1 (s)	14, 15, 16, 18
19		110.1 (s)	7, 20, 21
20	1.50 (s)	24.4 (q)	21
21	1.68 (s)	26.4 (q)	20

Table S7. ^{1}H and ^{13}C NMR data for 7-dehydro-7-deoxyaureothin (**6**) in $CDCl_3$.

Position	^{1}H (J_{Hz})	^{13}C (mult)	HMBC correlations (^{1}H to ^{13}C)
1			
2		162.0 (s)	
2a	4.06 (s)	55.7 (q)	2
3		99.9 (s)	
3a	1.86 (s)	7.3 (q)	2, 3, 4
4		181.2 (s)	
5		119.3 (s)	
5a	2.06 (s)	10.0 (q)	4, 5, 6
6		152.5 (s)	
7	6.52 (d 15.6)	117.6 (d)	5, 6, 8, 9
8	6.97 (d 15.6)	139.1 (s)	6, 7, 9, 9a, 10
9		135.5 (s)	
9a	2.12 (s)	14.5 (s)	8, 9, 10
10	6.32 (s)	140.3 (d)	8, 9, 9a, 11a, 12
11		138.9 (s)	
11a	2.12 (s)	19.5 (q)	10, 11, 12
12	6.54 (s)	130.5 (d)	10, 11, 11a, 13, 14, 15
13		144.3 (s)	
14,15	7.43 (d 8.6)	129.9 (d)	12, 14, 15, 16, 17, 18
16,18	8.19 (d 8.6)	123.8 (d)	13, 16, 17, 18
17		146.4 (s)	

Table S8. ^{1}H and ^{13}C NMR data for 2-pyrone-7-dehydro-7-deoxyaureothin (**7**) in $CDCl_3$.

Position	^{1}H (J_{Hz})	^{13}C (mult)	HMBC correlations (^{1}H to ^{13}C)
1			
2		1654.9 (s)	
3		111.2 (s)	
3a	2.04 (s)	10.4 (q)	2, 3, 4
4		168.1 (s)	
4a	3.78 (s)	60.2 (q)	
5		110.6 (s)	
5a	2.02 (s)	9.7 (q)	4, 5, 6
6		1532.9 (s)	
7	6.344 (d 15.6)	115.8 (d)	5, 6, 8, 9
8	7.20 (d 15.6)	139.6 (s)	
9		135.3 (s)	
9a	2.07 (s)	14.1 (s)	8, 9, 10
10	6.341 (s)	140.2 (d)	8, 9a, 11a, 12
11		139.1 (s)	
11a	2.09 (s)	19.2 (q)	10, 11, 12
12	6.50 (s)	130.1 (d)	10,11a, 13, 14, 15
13		144.2 (s)	
14,15	7.42 (d 8.4)	129.6 (d)	12, 14, 15, 16, 17, 18
16,18	8.17 (d 8.4)	123.5 (d)	13, 16, 17, 18
17		146.0 (s)	

SUPPLEMENTARY FIGURES

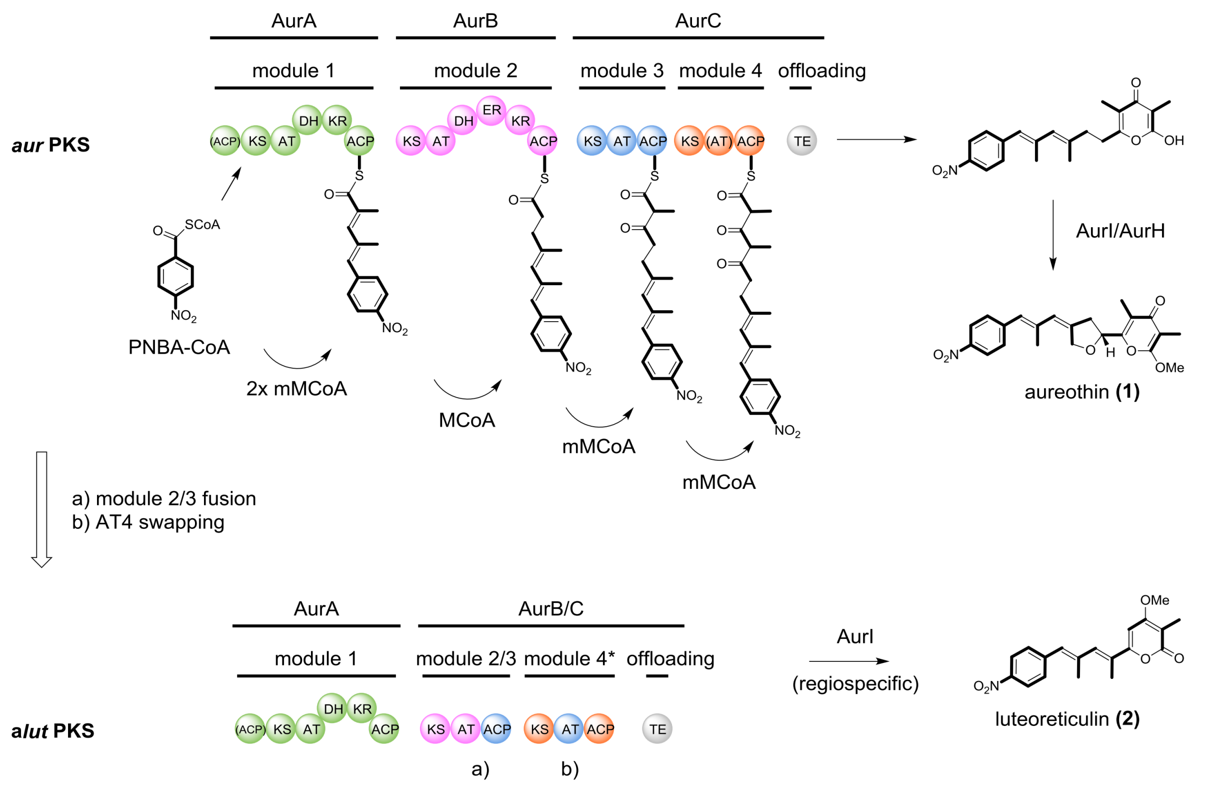

Figure S1. The artificial luteoreticulin PKS (*alut* PKS) engineered from the *aur* PKS by applying the co-linearity rule.[7] The *alut* PKS lacks the sequences between AT2 and ACP3, and the inactive AT4 is exchanged to AT3.

```
KR Domain type     LDD loop                 Catalytic region                    Finger print

AurB-KR2   B1   HTVGVVDDGVLETM VFRPKADGAWHLHELTRDR-DLAAFAVCSSVAGTLGSAAQANYAAAN
Tyl1       B1   HTAGILDDAVIDTL VRGAKVCGAELLHQLTADIKGLDAFVLFSSVTGTWGNAGQGAYAAAN
Ave1       B1   HTAGILDDATLTNL VLRAKAHSAHLLHQLTQHT-PLTAFVLYSSAAATFGAPGQANYAAAN
Ave7       B1   HAAGVLDDATIASL VFAAKVDAALLLDELTRGM-ELSAFVLFSSAAGILGSAGQGNYAAAN  (LDD, no P)
Ave9       B1   HAAGVLDDATIASL VFAAKVDAALLLDELTRGM-ELSAFVLFSSAAGILGSAGQGNYAAAN
Rap10      B1   HTAGVLDDGVVESL VLRPKADGAWNLHELTRDI-DLAAFVMYSSAAGVLGSAGQGNYAVAN
Nys3       B1   HTAGVVDDGVLGSL VLRPKADAAWHLHEATRHL-DLDAFVLFSSVAATLGSPGQANYAAGN
Nys12      B1   HTAGVVEDHVVDAL VLRAKTVAAHHLHELTADL-DLAAFVLFSSTAGVLGAAGQGNYAAAN

Pik1       B2   HTAGALDDGIVDTL AHRAKAVGASVLDELTRDL-DLDAFVLFSSVSSTLGIPGQGNYAPHN
Lkm1       B2   HTAATLDDGTLDTL AGRAKTLGARNLHELTADA-PLTAFVLFSSFASAFGAPGLGCYAPGN  (LDD, P)
Ery1       B2   HAAATLDDGTVDTL ASRAKVLGARNLHELTREL-DLTAFVLFSSFASAFGAPGLGGYAPGN

Ery2       A1   HAAGLPQQVAINDM VVAAKAGGAVHLDELCSDA---ELFLLFSSGAGVWGSARQGAYAAGN  (no LDD; W; no H)
Pik5       A1   HTAGAPGGDPLDVT ILGAKTSGAEVLDDLLRGT-PLDAFVLYSSNAGVWGSGSQGVYAAAN

Amph1      A2   HTAAVIELAALADT VVHAKVTGARILDELLDDE-ELDDFVLYSSTAGMWGSGVHAAYVAGN  (no LDD; W; H)
Nys1       A2   HAAAAIELSALADT VVHAKVTGARILDELLDDA-ELDDFVLYSSTAGMWGSGVHAAYVAGN
                     ...           *                        *    .    .  * .
```

Figure S2. Sequence alignment of KR domains. The KR2 domain in aureothin PKS was aligned with other known KR domains by program Clustal Omega.[8] Tyl: tylosin, Ave: avermectin, Rap: rapamycin, Pik: pikromycin, Lkm: lankamycin, Ery: erythromycin, Amph: amphotericin, Nys: nystatin. The conserved lysine, tyrosine, and serine (indicated by star) aid in binding and reducing polyketide intermediates. Fingerprint residues (indicated by dot) are hypothesized to help guide polyketide intermediates into the active site. Normally, A-type KRs generate an L-orientation hydroxyl group, B-type KRs generate a D-orientation hydroxyl group; and 1 if the a-substituent is not epimerized and 2 if the a-substituent is epimerized.[9]

A

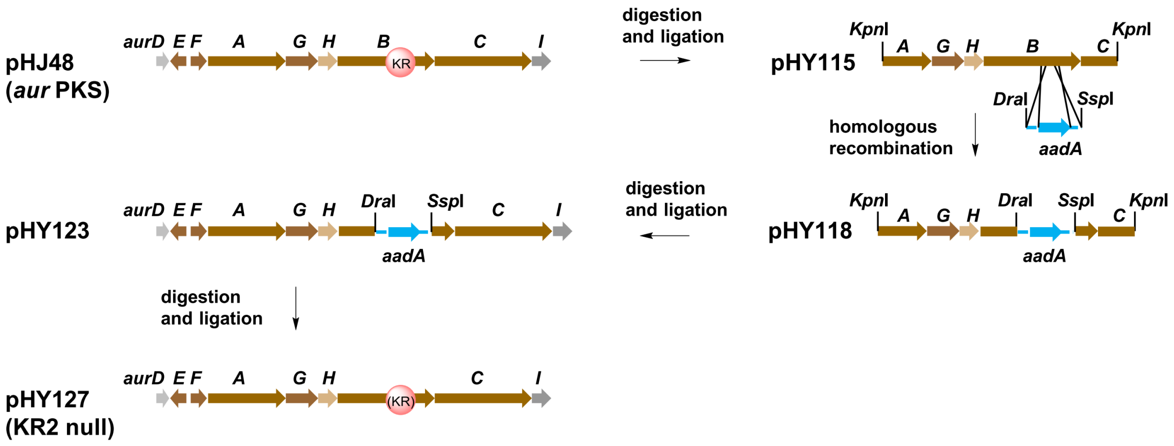

B

pHJ48	GCC GCC CAG GCC AAC TAC GCG GCC GCC AAC GCC TTC CTC GAC
	A A Q A N Y A A A N A F L D
	(*Dra*I) (*Ssp*I)
pHY123	GCC GCC CAG GCC AAC TTT AAA --- AAA TAT TGC CGC CAA CGC CTT CCT CGA C
	A A Q A N F K --- K Y C R Q R L P R
pHY127	GCC GCC CAG GCC AAC TTT ATT GCC GCC AAC GCC TTC CTC GAC
	A A Q A N F I A A N A F L D

Figure S3. Schematic strategy to generate KR2 inactive mutant. A) Workflow to generate the KR2 inactive mutant *S. albus*::pHY127 (KR2 null). B) Nucleotide/amino acid sequences of the original and mutated sites. The mutation sites induced by restriction enzyme *Dra*I and *Ssp*I are highlighted in red.

A

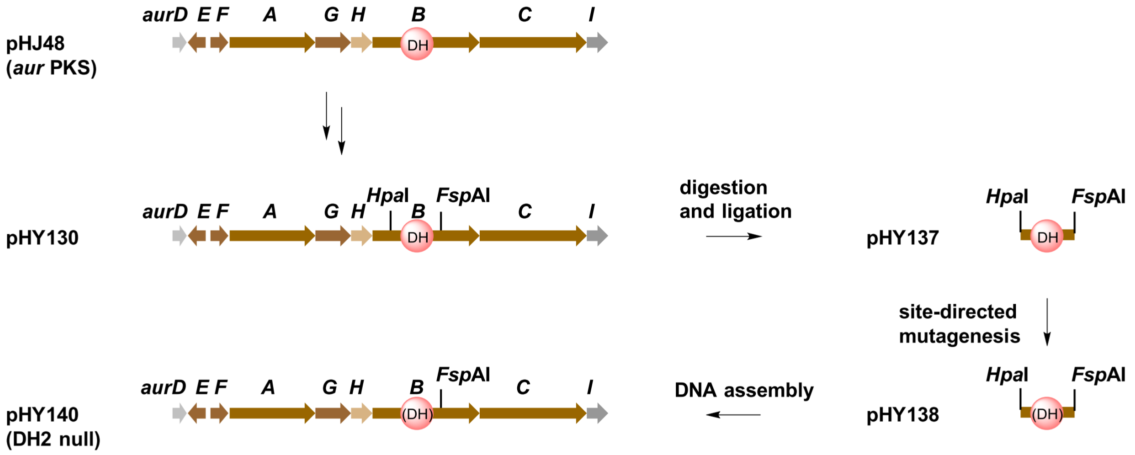

B

pHJ48 **CTG GCC GAC CAC GTC GTG CTC GGC TCC ACG CTC GTC CCG**
L A D H V V L G S T L V P

pHY140 **CTG GCC GAC <u>TTC</u> GTC GTG CTC GGC TCC ACG CTC GTC CCG**
L A D F V V L G S T L V P

Figure S4. Schematic strategy to generate DH2 inactive mutant. A) Workflow to generate the DH2 inactive mutant *S. albus*::pHY140 (DH2 null) by site-directed mutagenesis. B) Nucleotide/amino acid sequences of the original and mutated sites. The mutation site is highlighted in red.

A

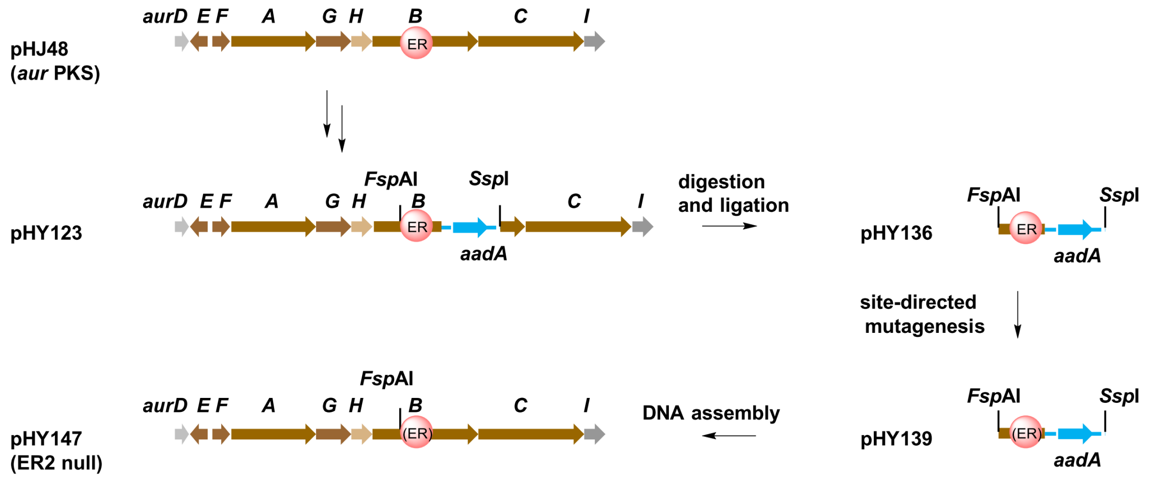

B

**pHJ48**	**GCG GCG GGC GGC GTC GGC ATG GCC GCC GTC**<br>**A A G G V G M A A V**
**pHY147**	**GCG GCG AGC CCC GTC GGC ATG GCC GCC GTC**<br>**A A S P V G M A A V**

Figure S5. Schematic strategy to generate ER2 inactive mutant. A) Workflow to generate the ER2 inactive mutant *S. albus*::pHY142 (ER2 null) by site-directed mutagenesis. B) Nucleotide/amino acid sequences of the original and mutated sites. The mutation site is highlighted in red.

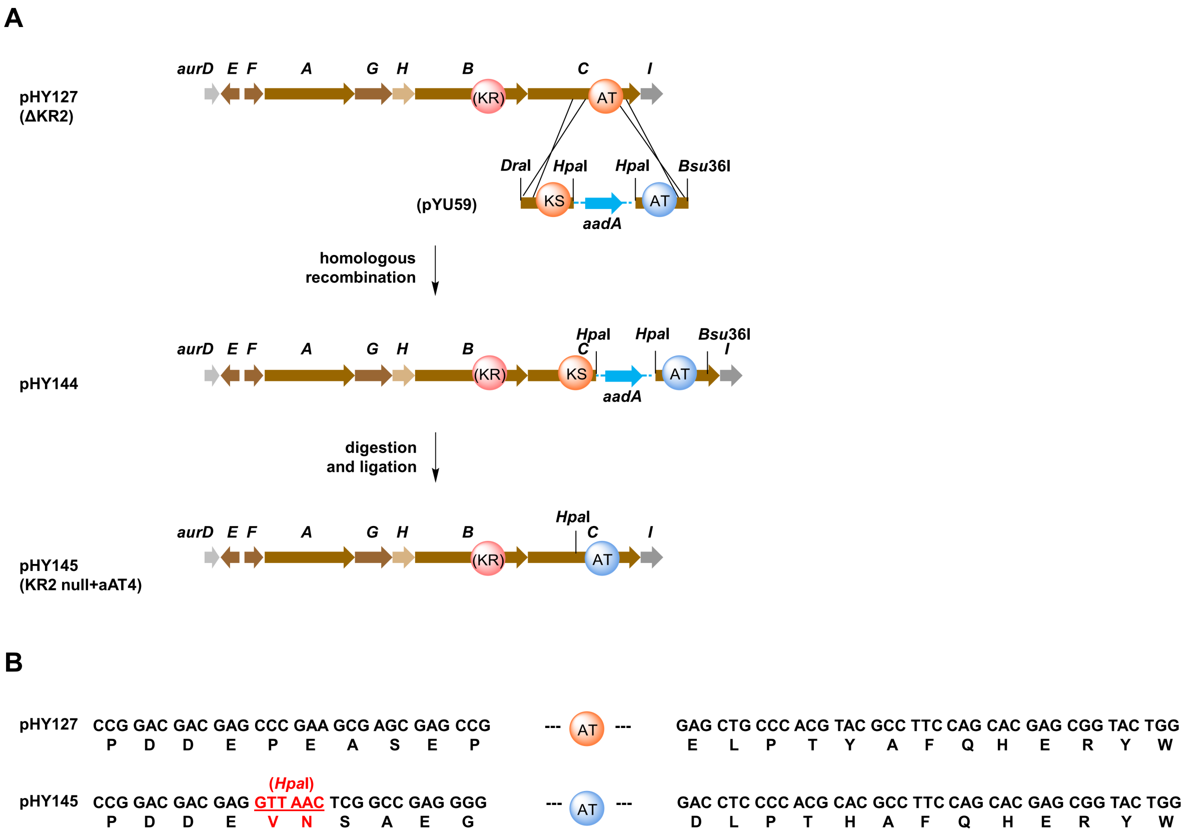

Figure S6. Schematic strategy to generate KR2 inactive and AT4 exchange mutant. A) Workflow to generate the mutant *S. albus*::pHY145 (KR2 null+aAT4). B) Nucleotide/amino acid sequences of the original and mutated sites. The mutation sites induced by restriction enzyme *Hpa*I.

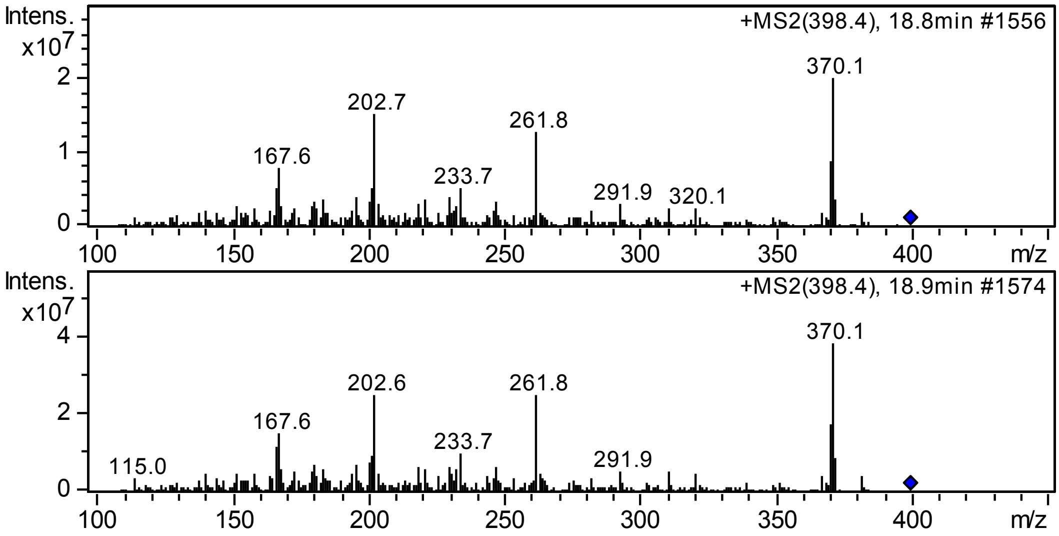

Figure S7. LC-MS/MS analysis of aureothin (**1**). MS/MS spectra at *m/z* 398.4 $(M+H)^+$ for aureothin obtained from the EtOAc extracts of the cultures *S. albus*::pHJ48 (upper) and *S. albus*::pHY127 (lower).

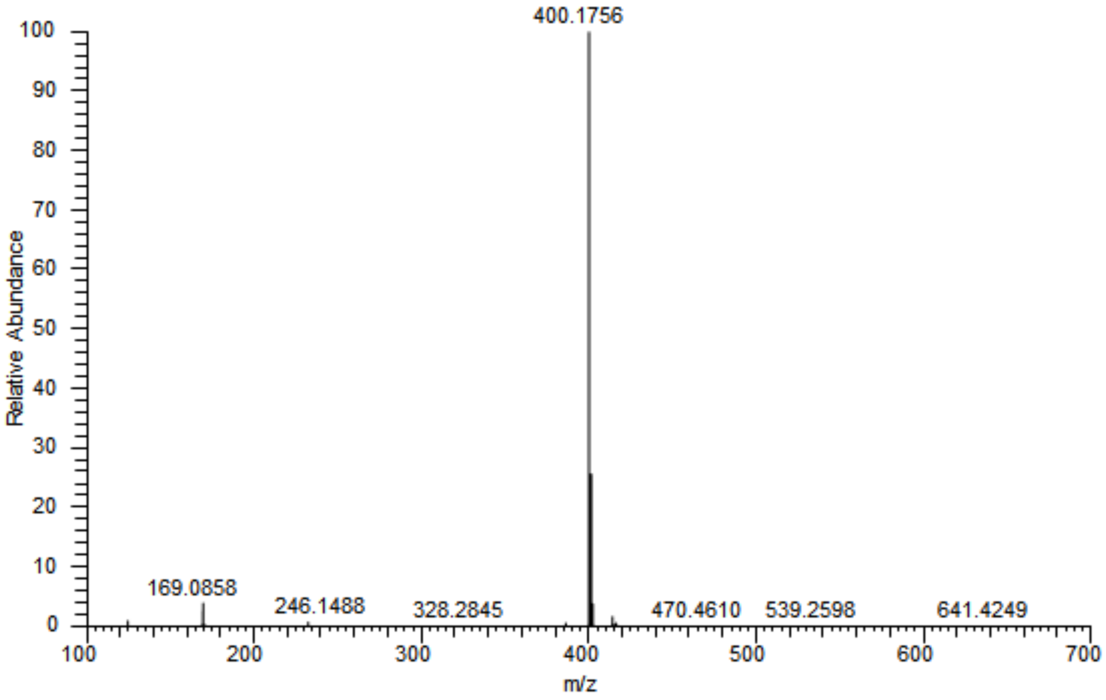

Figure S8. High-resolution MS spectrum of **3** in positive ion mode.

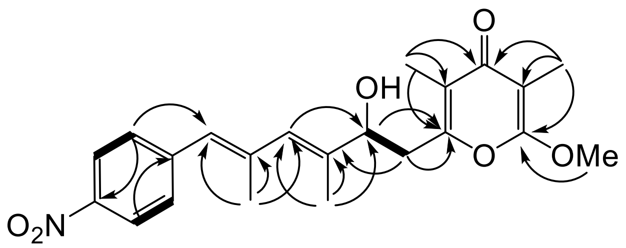

Figure S9. Selected ^{1}H-^{1}H COSY (bold lines) and HMBC (arrows) correlations of **3**.

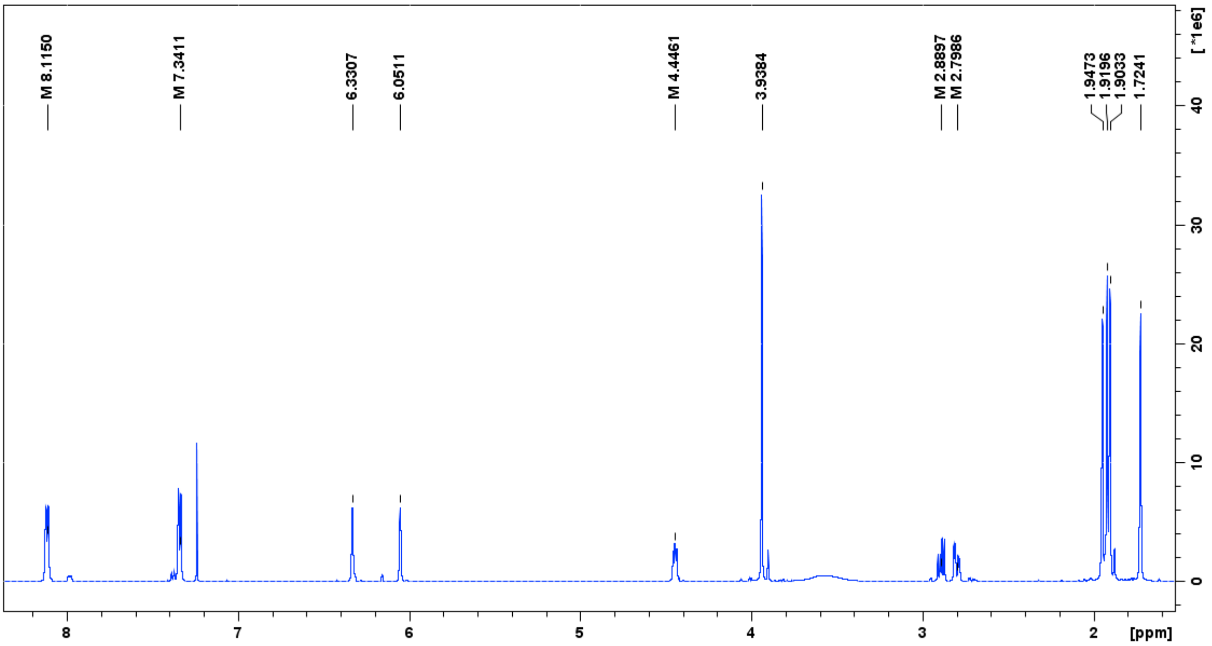

Figure S10. ^{1}H NMR spectrum of 8-hydroxy-7-deoxyaureothin (**3**) in $CDCl_3$ at 300 K.

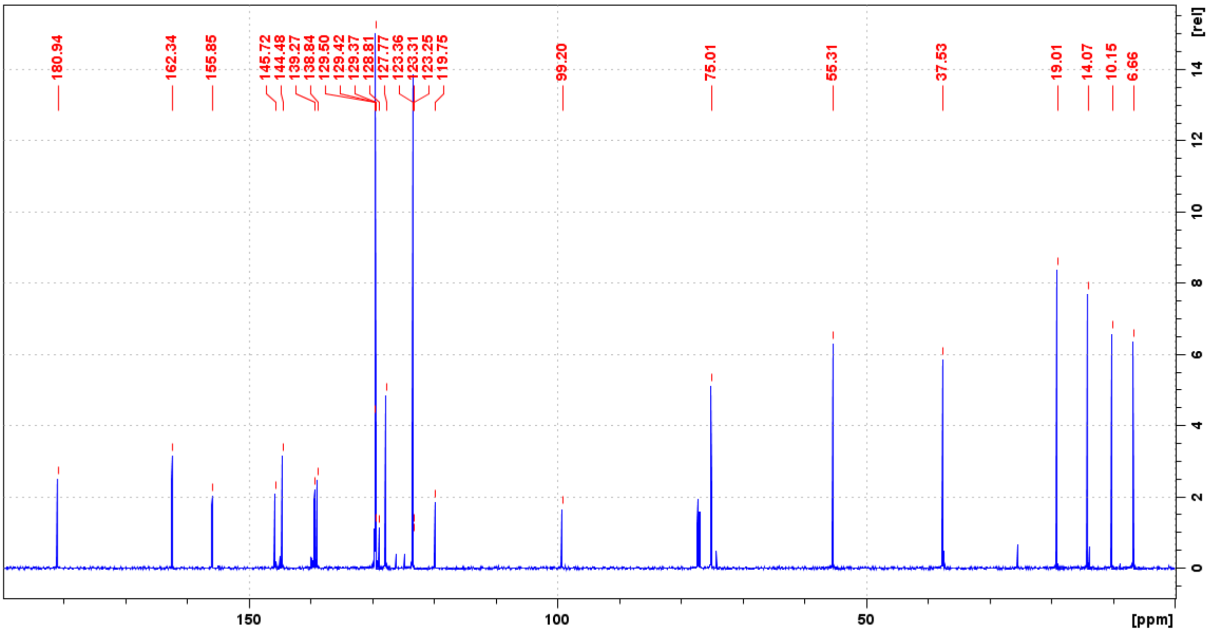

Figure S11. ^{13}C NMR spectrum of 8-hydroxy-7-deoxyaureothin (**3**) in $CDCl_3$ at 300 K.

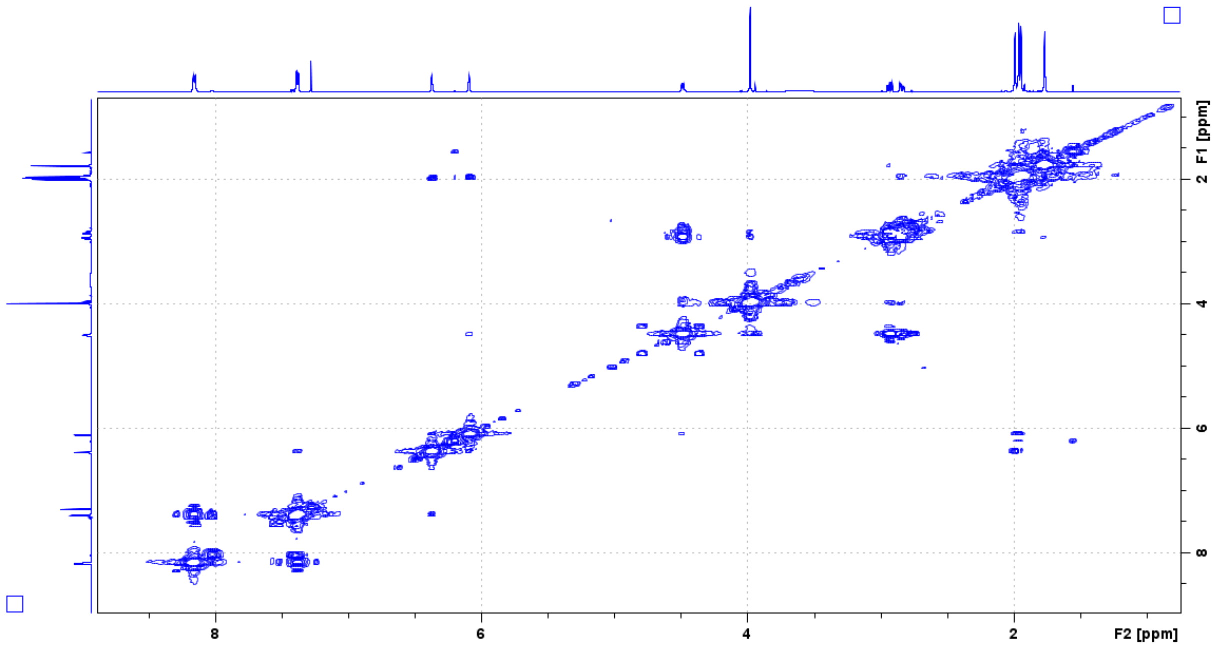

Figure S12. ^{1}H-^{1}H COSY spectrum of 8-hydroxy-7-deoxyaureothin (**3**) in $CDCl_3$ at 300 K.

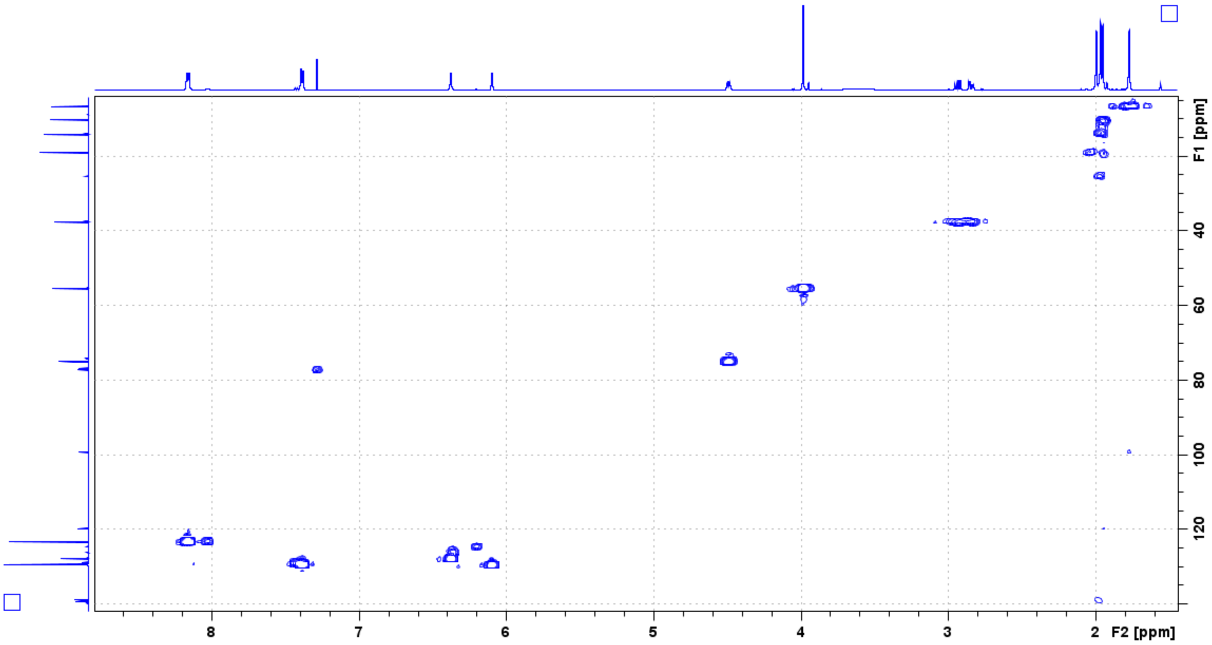

Figure S13. HSQC spectrum of 8-hydroxy-7-deoxyaureothin (**3**) in $CDCl_3$ at 300 K.

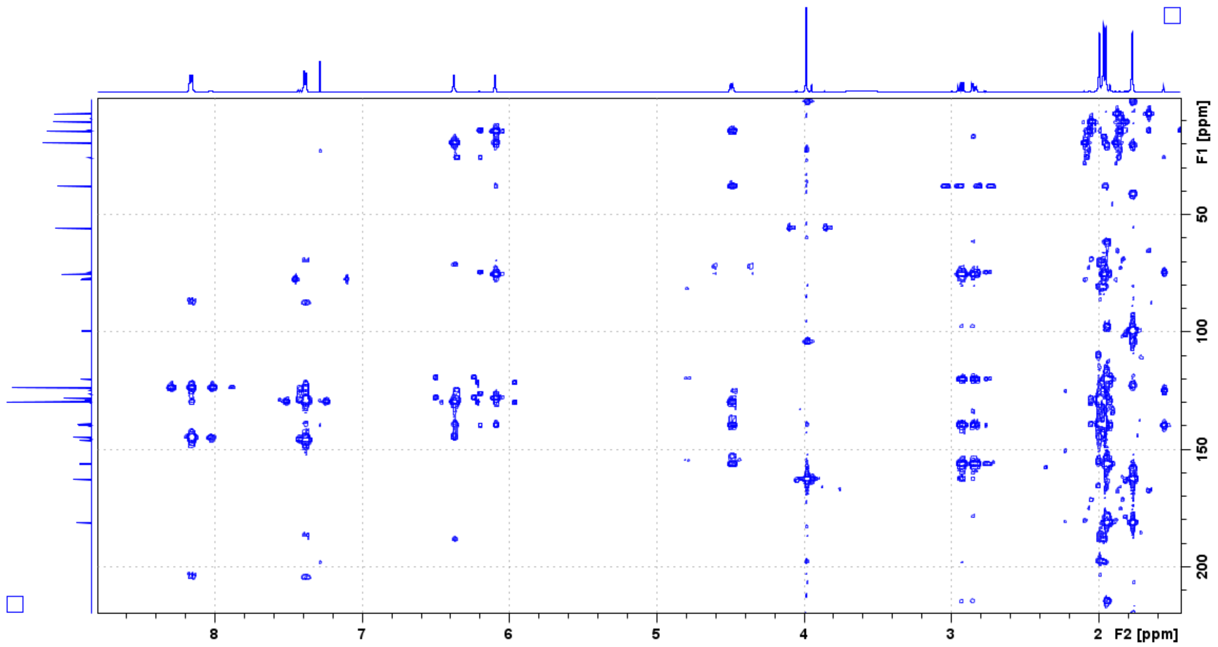

Figure S14. HMBC spectrum of 8-hydroxy-7-deoxyaureothin (**3**) in $CDCl_3$ at 300 K.

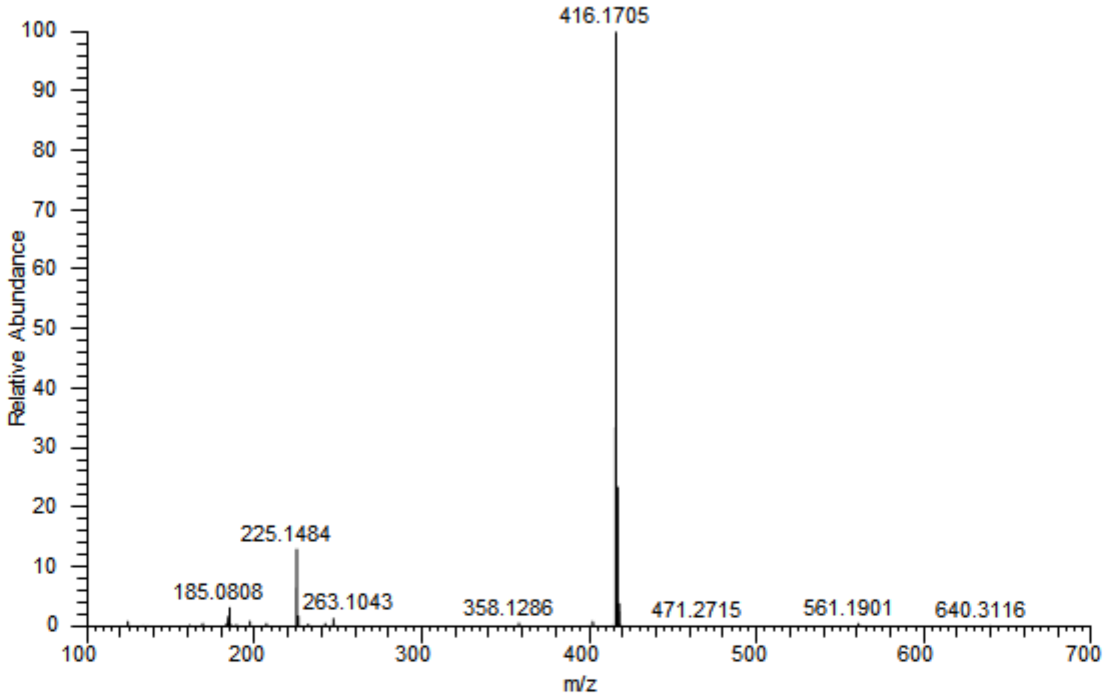

Figure S15. High-resolution MS spectrum of **4** in positive ion mode.

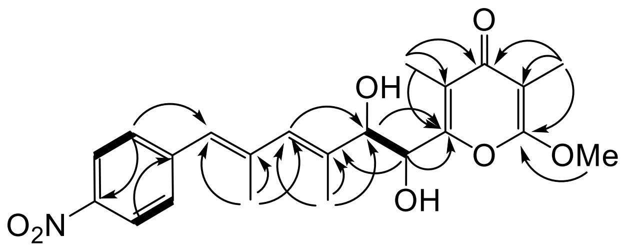

Figure S16. Selected ^{1}H-^{1}H COSY (bold lines) and HMBC (arrows) correlations of **4**.

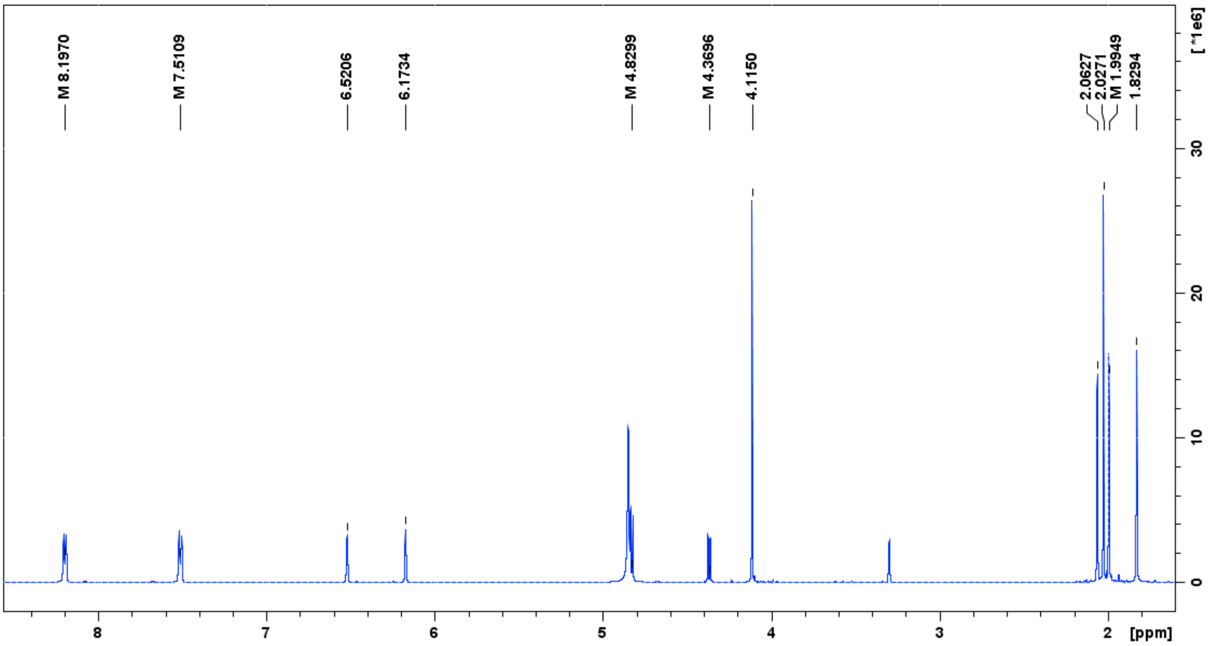

Figure S17. ^{1}H NMR spectrum of 7,8-dihydroxy-9-deoxyaureothin (**4**) in CD_3OD at 300 K.

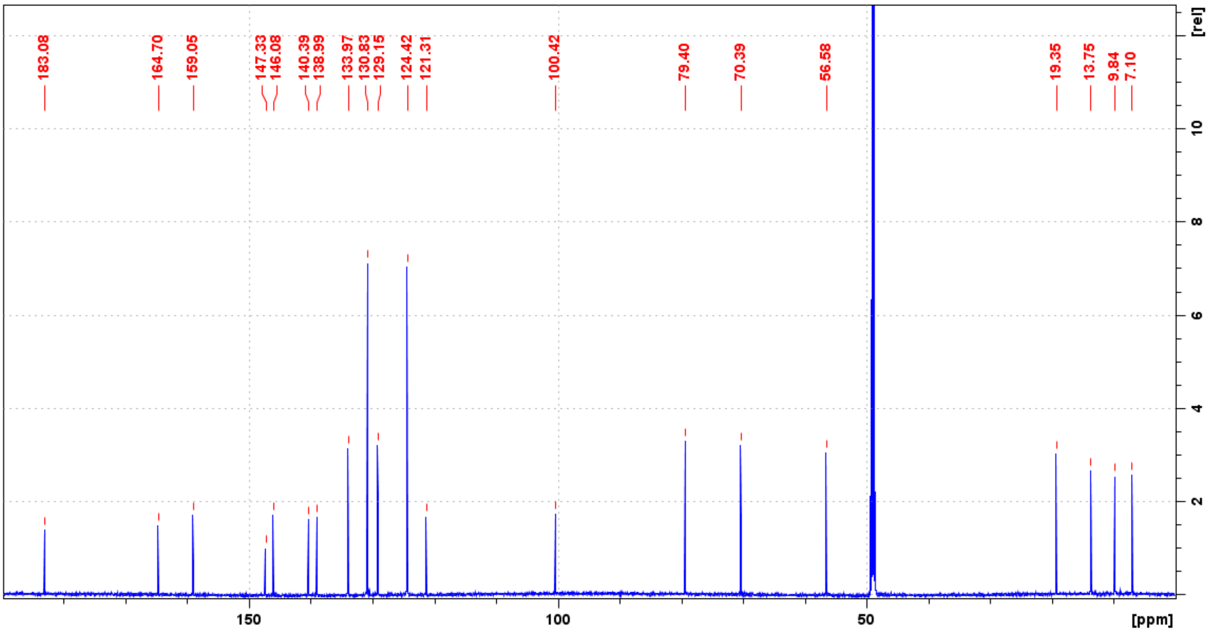

Figure S18. ^{13}C NMR spectrum of 7,8-dihydroxy-9-deoxyaureothin (**4**) in CD_3OD at 300 K.

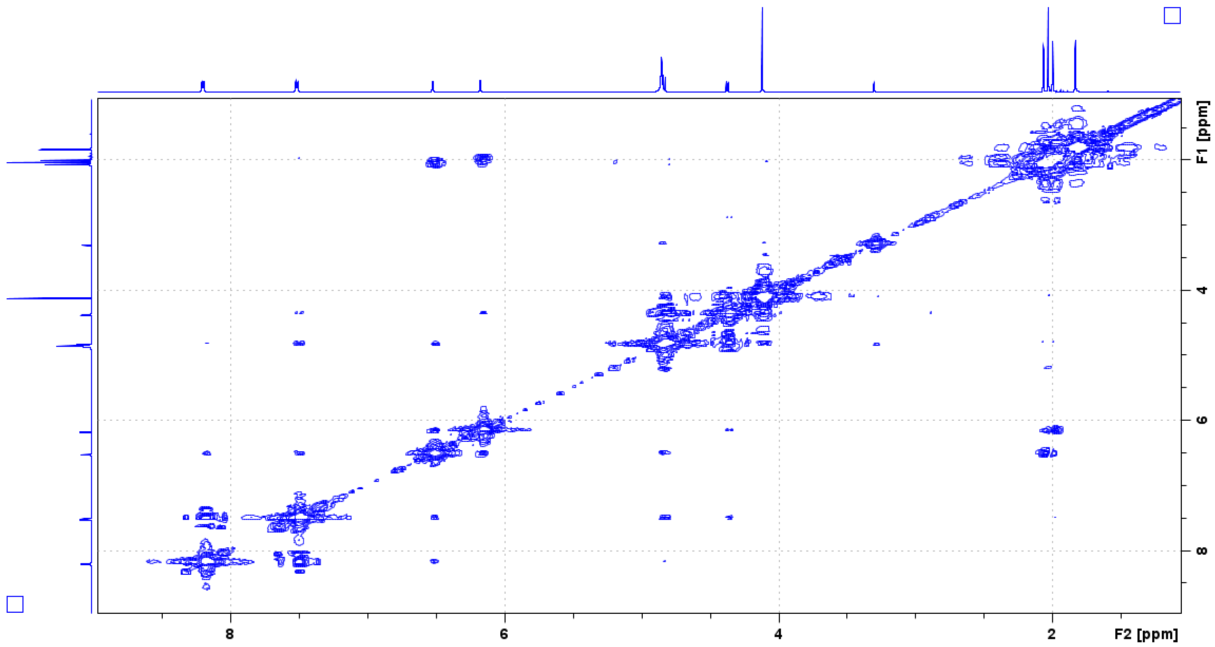

Figure S19. ^{1}H-^{1}H COSY spectrum of 7,8-dihydroxy-9-deoxyaureothin (**4**) in CD_3OD at 300 K.

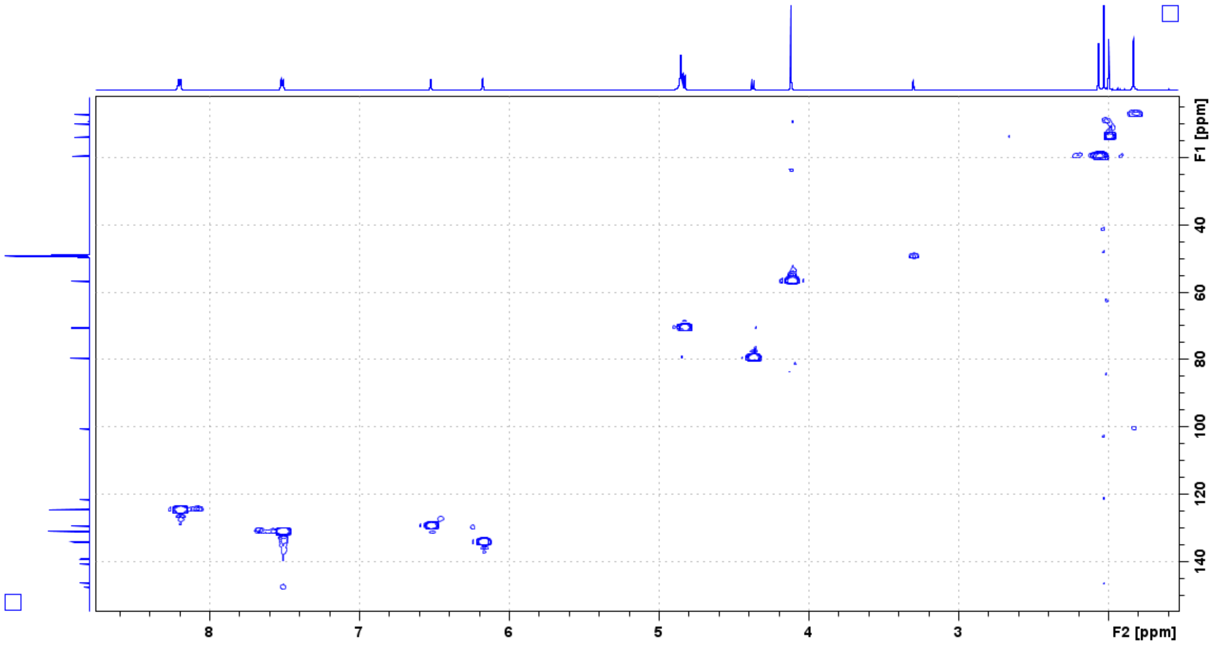

Figure S20. HSQC spectrum of 7,8-dihydroxy-9-deoxyaureothin (**4**) in CD_3OD at 300 K.

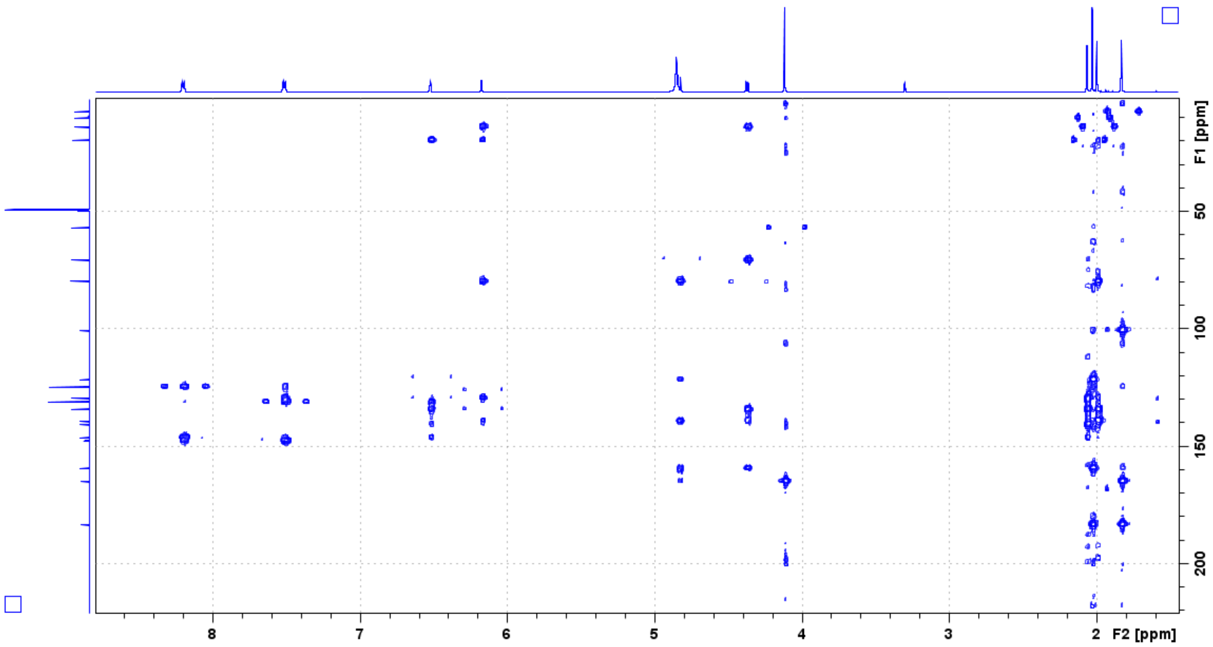

Figure S21. HMBC spectrum of 7,8-dihydroxy-9-deoxyaureothin (**4**) in CD_3OD at 300 K.

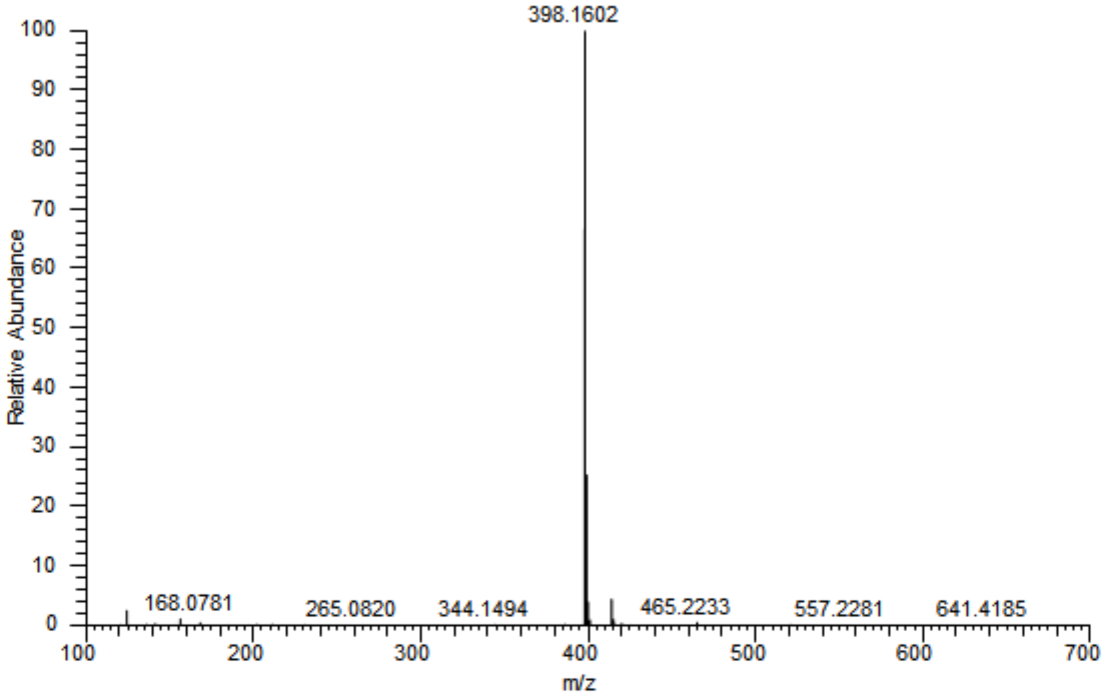

Figure S22. High-resolution MS spectrum of **5** in positive ion mode.

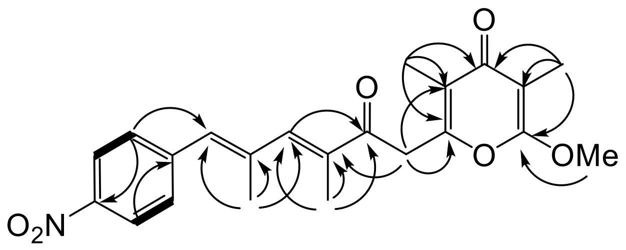

Figure S23. Selected ^1H-^1H COSY (bold lines) and HMBC (arrows) correlations of **5**.

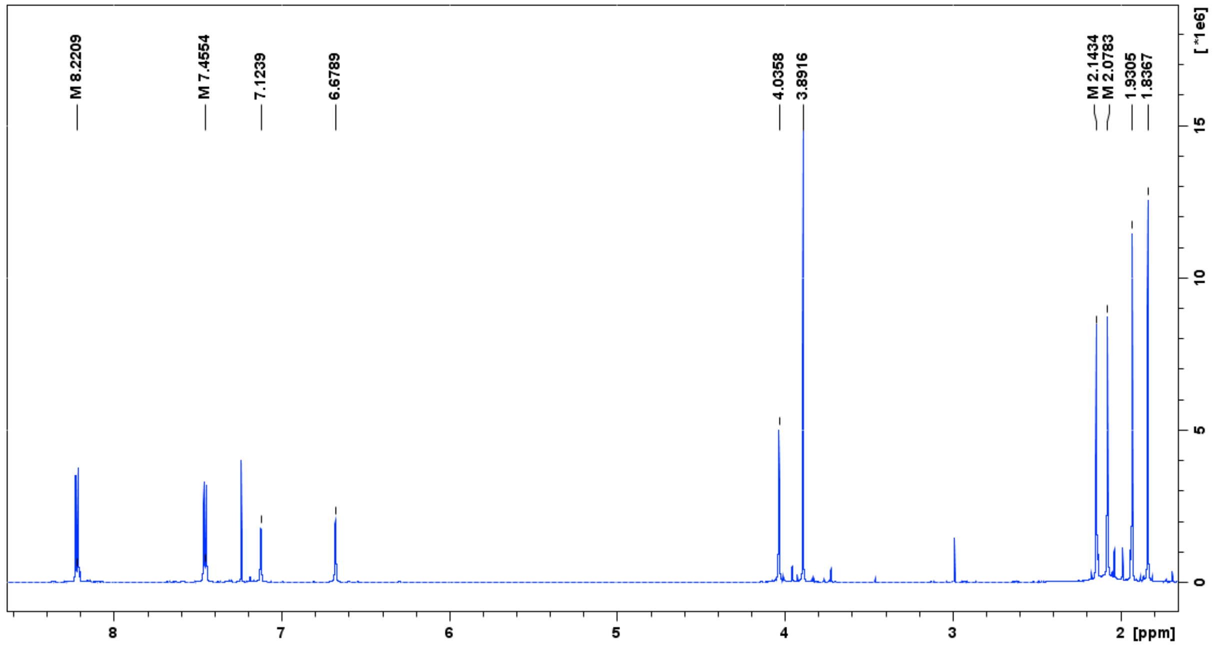

Figure S24. ^{1}H NMR spectrum of 8-oxo-7-deoxyaureothin (**5**) in $CDCl_3$ at 300 K.

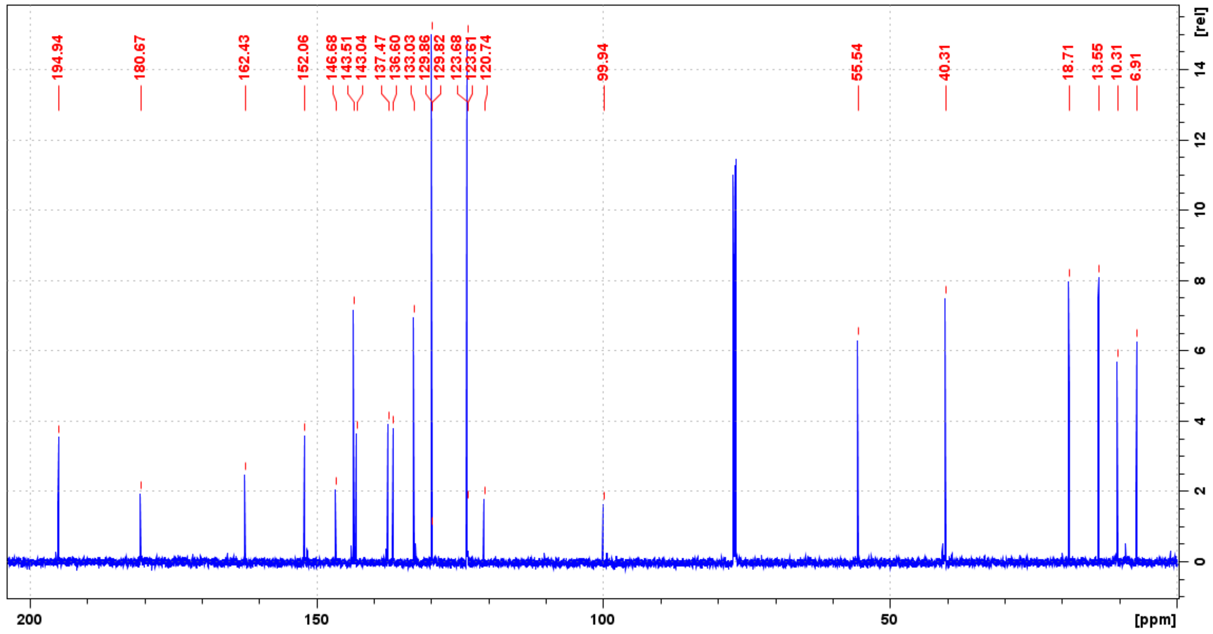

Figure S25. ^{13}C NMR spectrum of 8-oxo-7-deoxyaureothin (**5**) in $CDCl_3$ at 300 K.

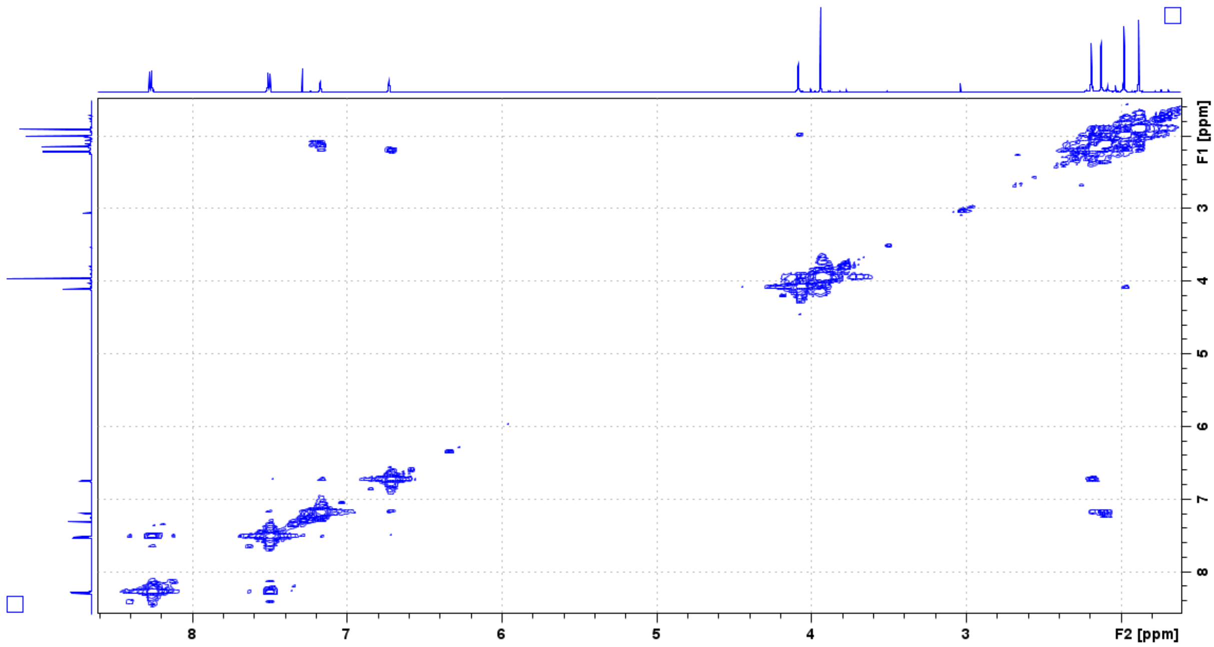

Figure S26. ^{1}H-^{1}H COSY spectrum of 8-oxo-7-deoxyaureothin (**5**) in $CDCl_3$ at 300 K.

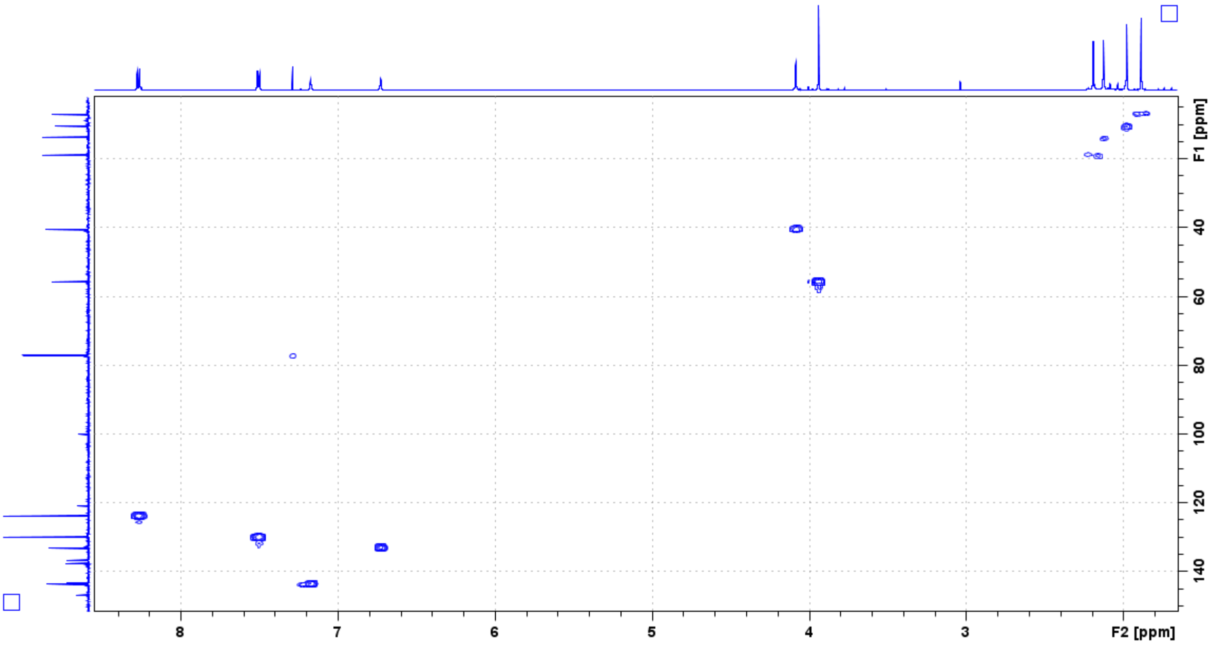

Figure S27. HSQC spectrum of 8-oxo-7-deoxyaureothin (**5**) in $CDCl_3$ at 300 K.

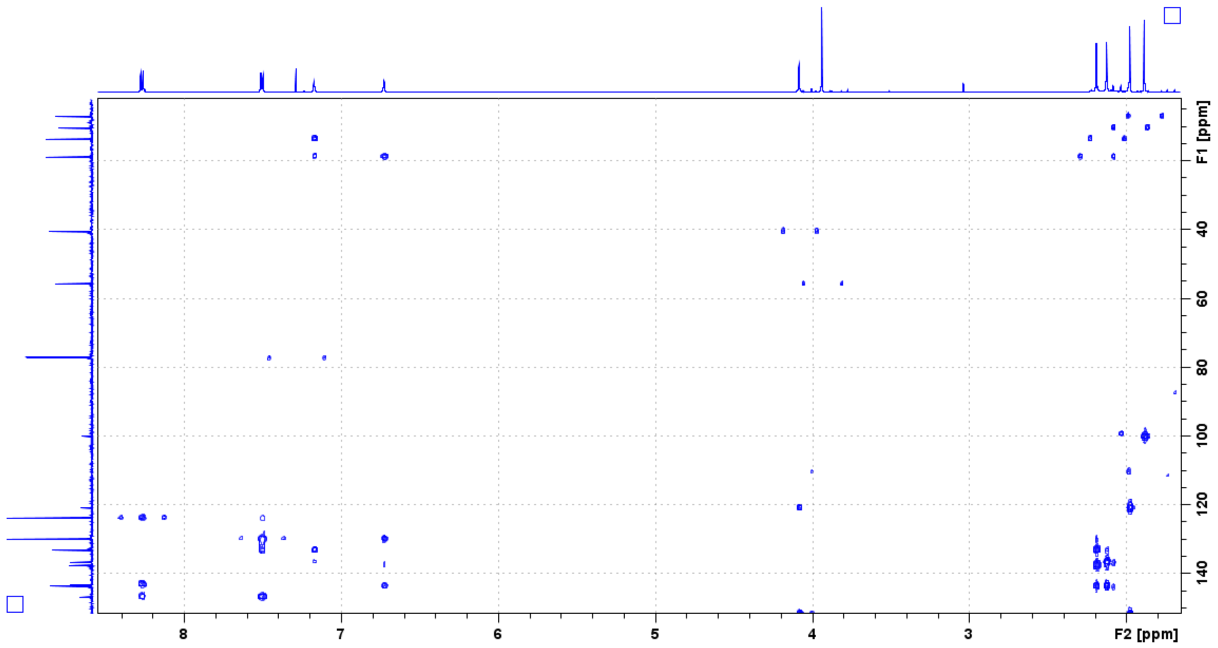

Figure S28. HMBC spectrum of 8-oxo-7-deoxyaureothin (**5**) in $CDCl_3$ at 300 K.

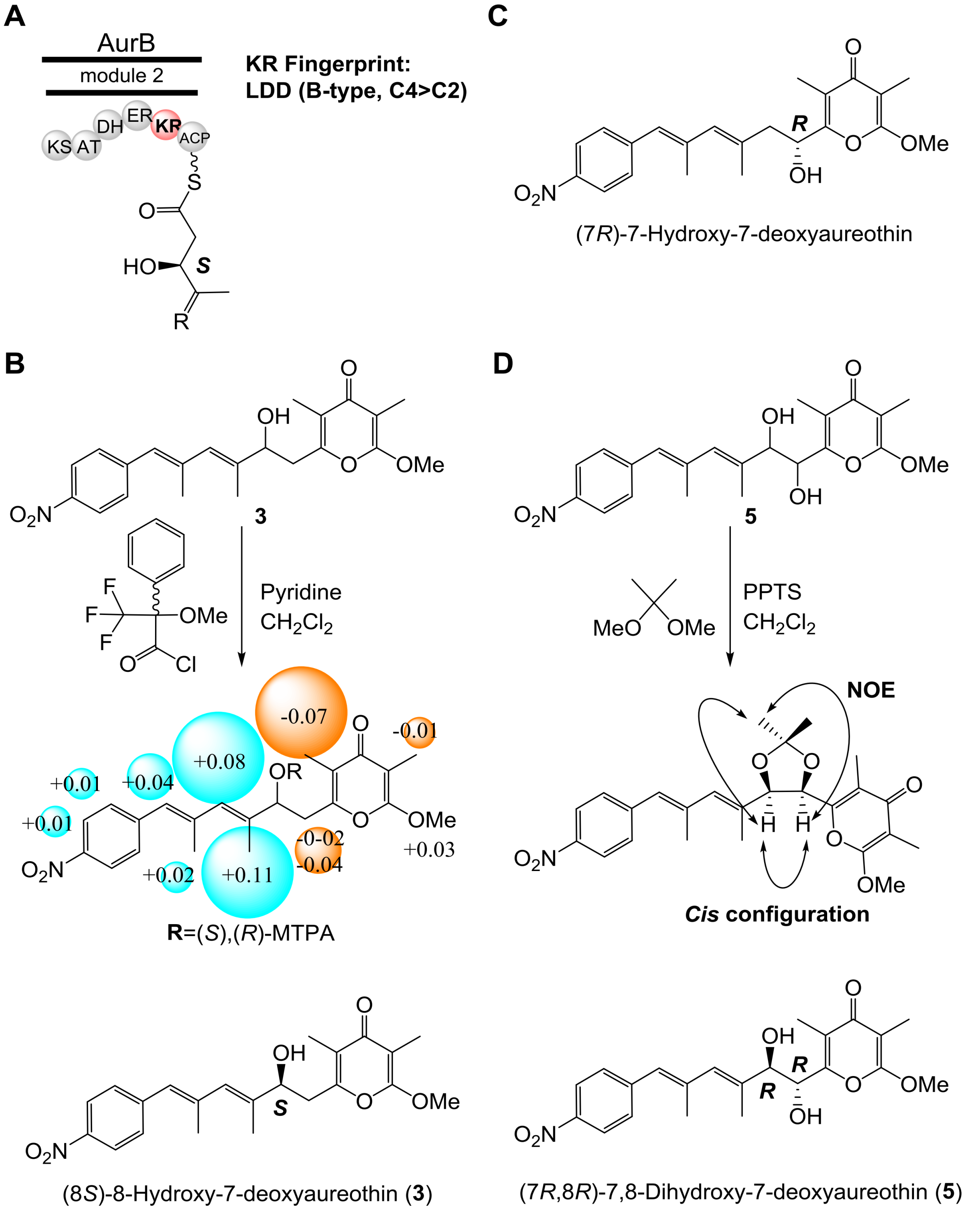

Figure S29. Determination of the absolute configuration of **3** and **4**. **A**) KR fingerprinting analysis of amino acid sequence[9] and intermediate structure[10] predicted that C8 of **3** has *S* configuration. (Figure S2) **B**) The δ_S-δ_R (ppm) values obtained from (*S*) and (*R*)-MTPA ester of **3** revealed that C8 of **3** has *S* configuration. **C**) AurH oxidized C7 as *S* configuration.[3] **D**) The NOESY corelations (NOE, arrows) obtained from acetonide of **4** showed that diol (C7,8) is *cis* configuration.

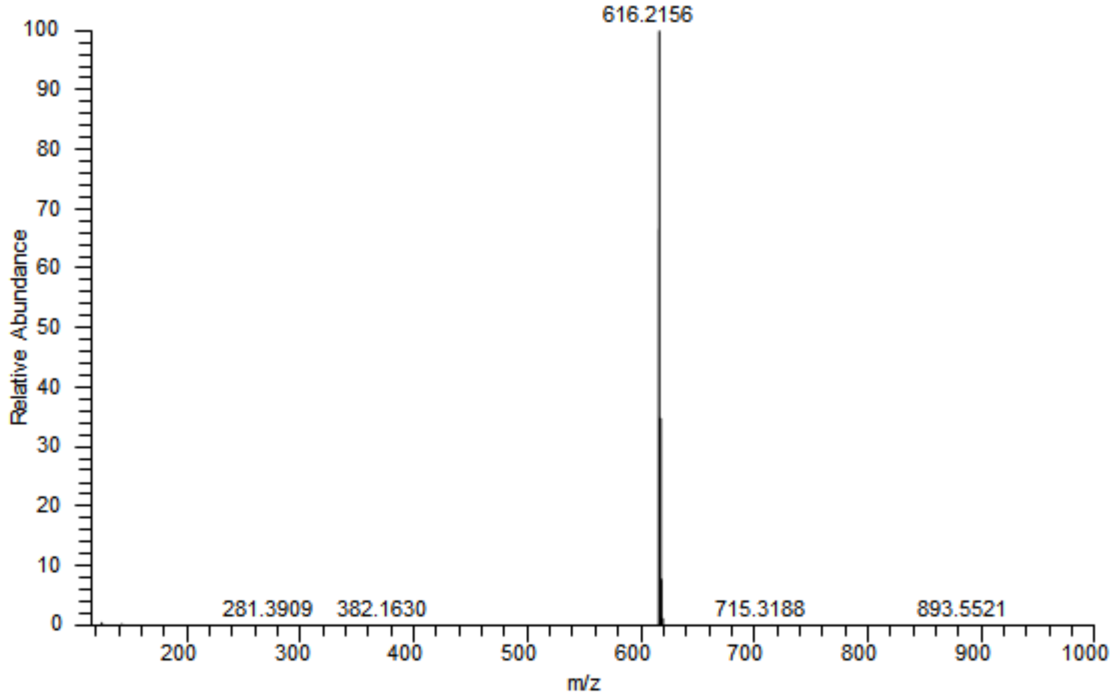

Figure S30. High-resolution MS spectrum of (*S*)-MTPA ester of **3** in positive ion mode.

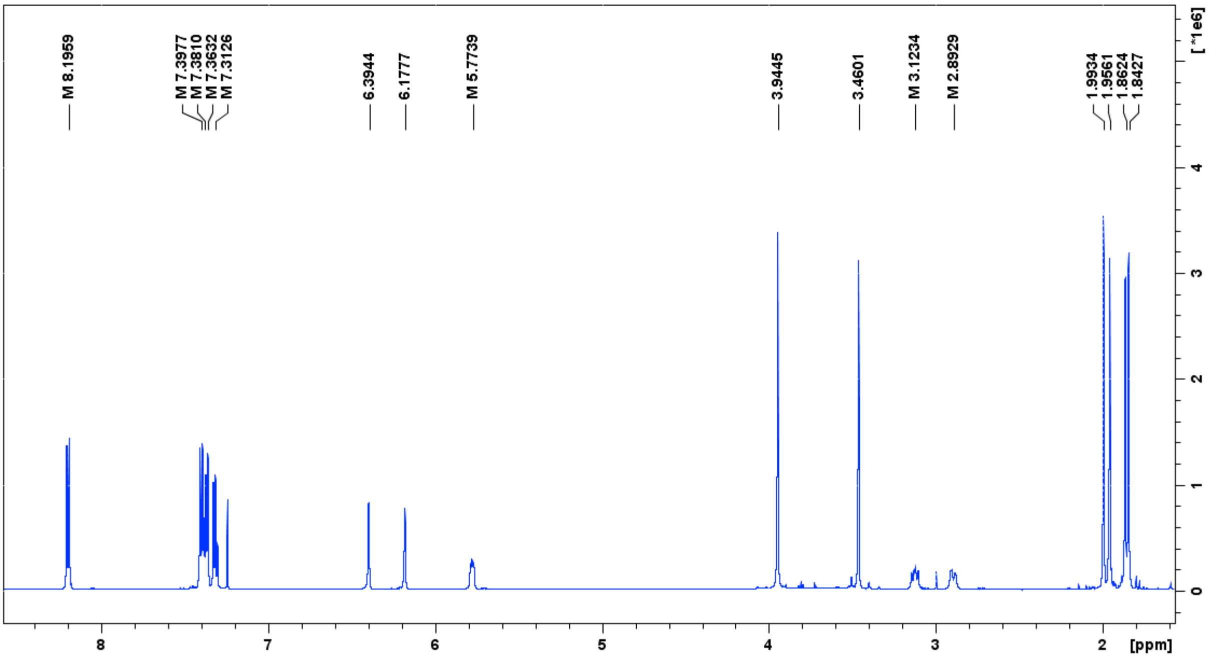

Figure S31. ^{1}H NMR spectrum of (*S*)-MTPA ester of **3** in $CDCl_3$ at 300 K.

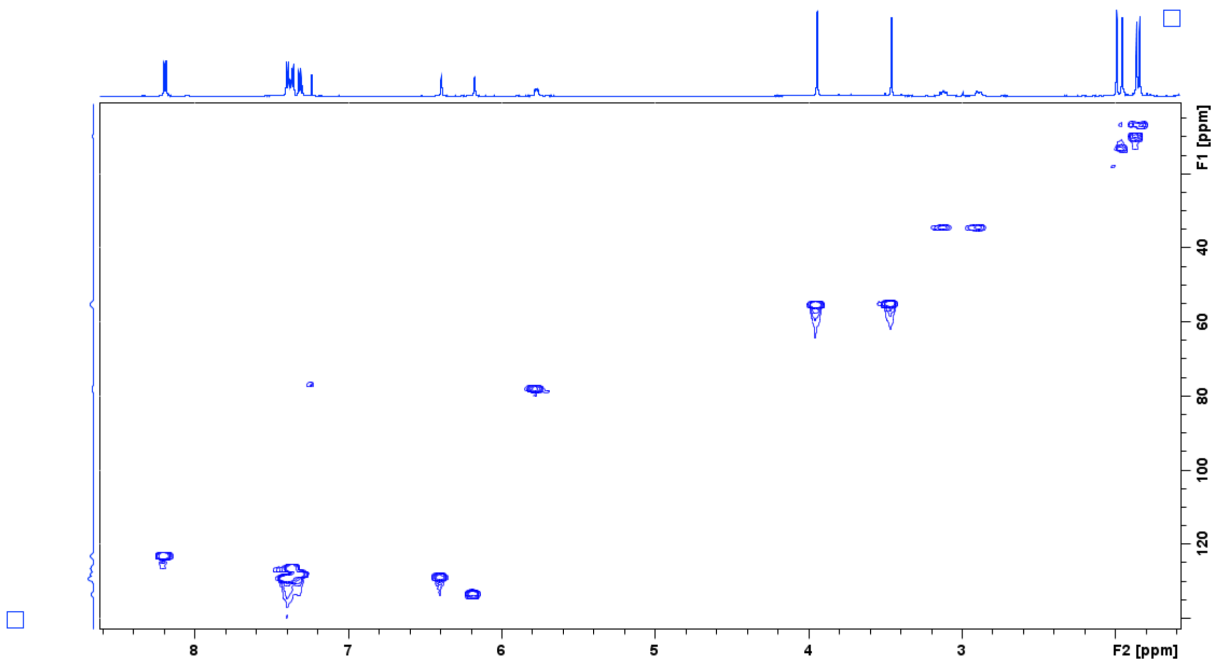

Figure S32. HSQC spectrum of (*S*)-MTPA ester of **3** in $CDCl_3$ at 300 K.

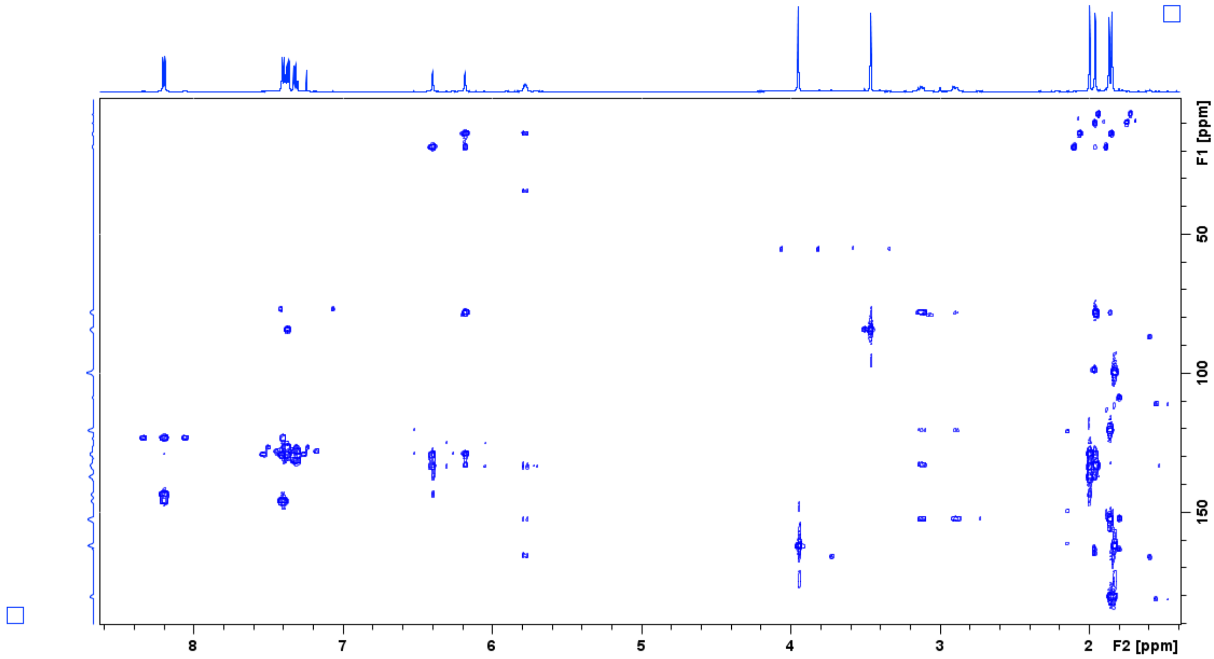

Figure S33. HMBC spectrum of (*S*)-MTPA ester of **3** in $CDCl_3$ at 300 K.

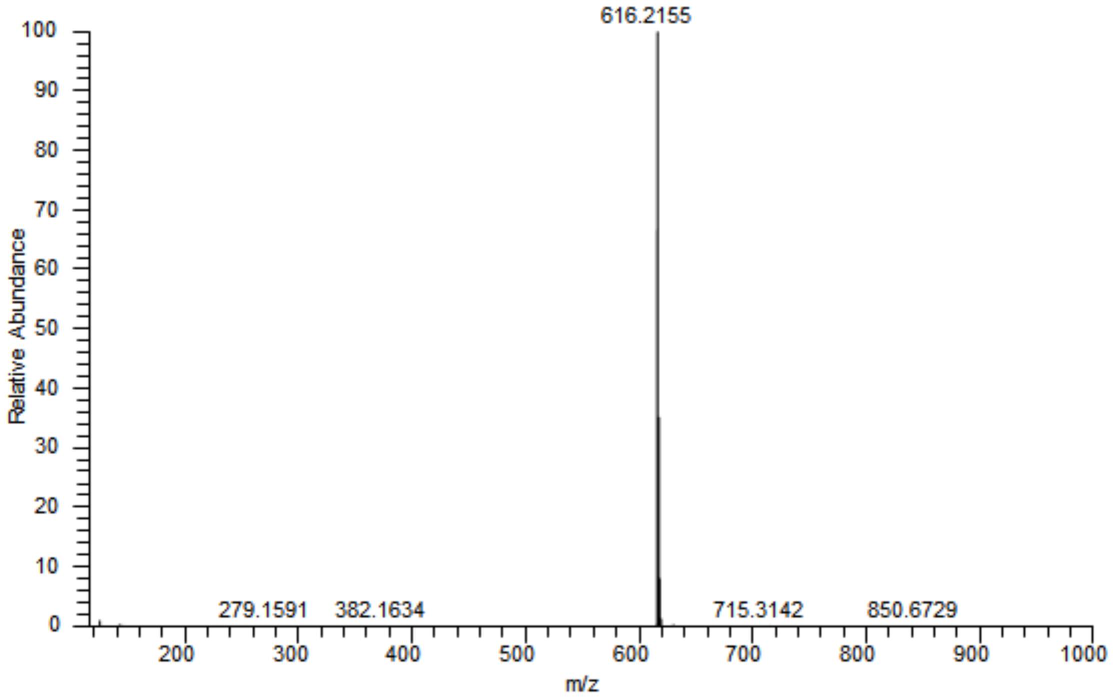

Figure S34. High-resolution MS spectrum of (*R*)-MTPA ester of **3** in positive ion mode.

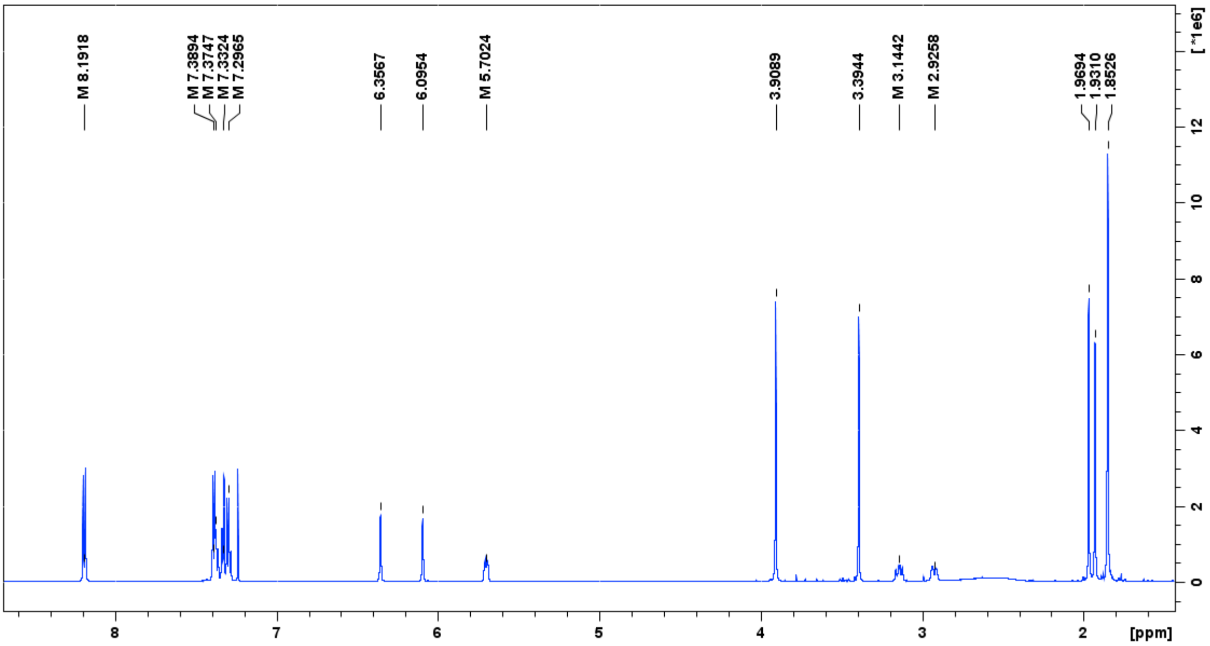

Figure S35. ^{1}H NMR spectrum of (*R*)-MTPA ester of **3** in $CDCl_3$ at 300 K.

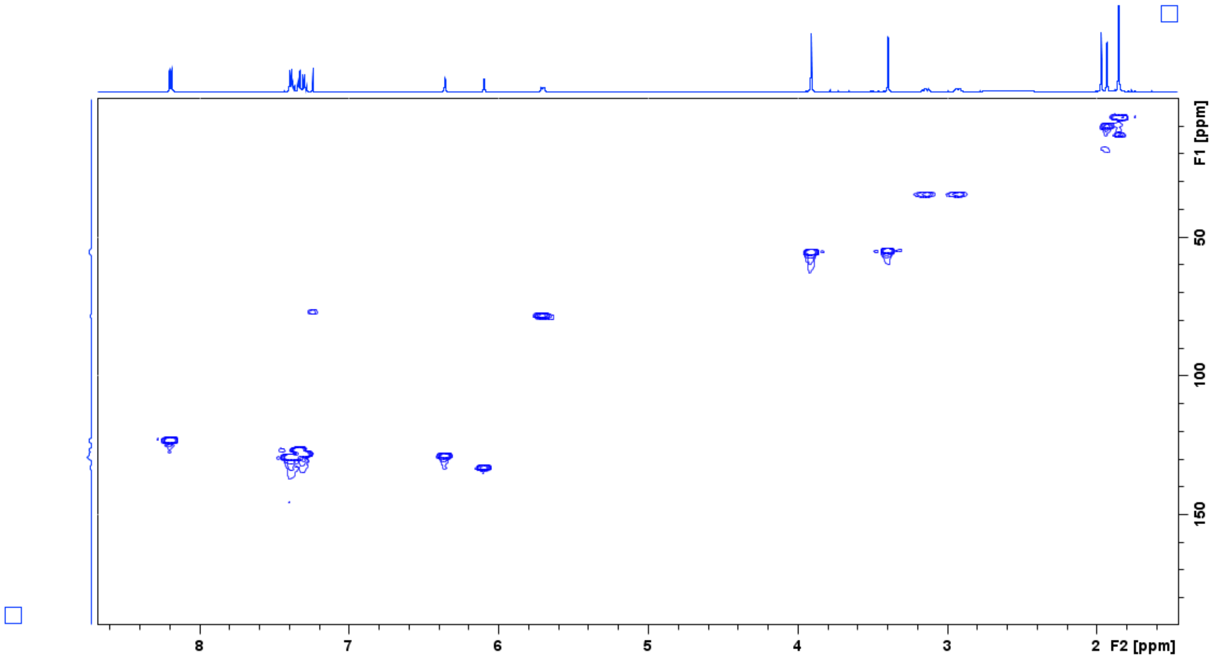

Figure S36. HSQC spectrum of (*R*)-MTPA ester of **3** in $CDCl_3$ at 300 K.

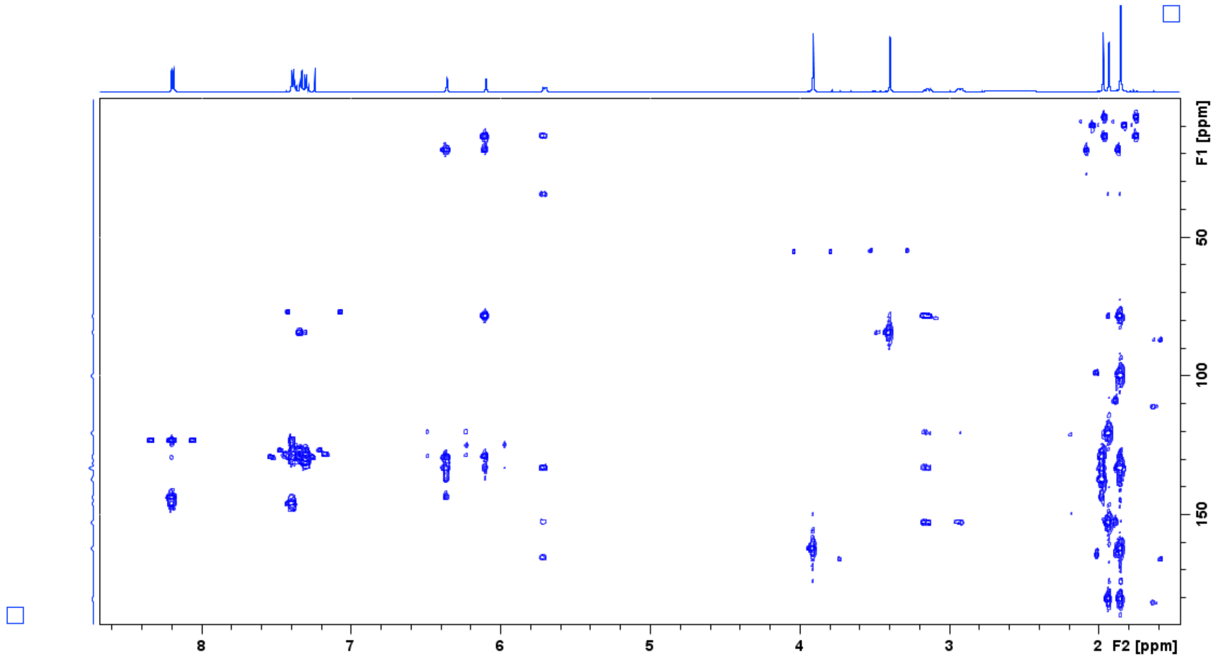

Figure S37. HMBC spectrum of (*R*)-MTPA ester of **3** in $CDCl_3$ at 300 K.

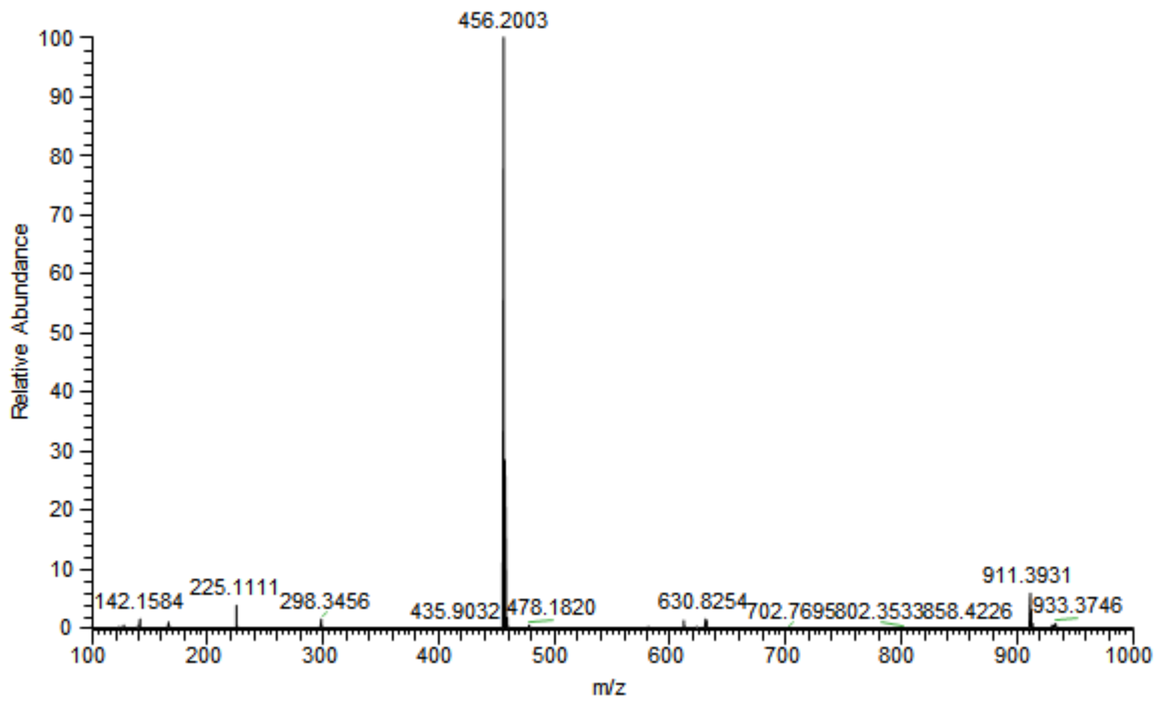

Figure S38. High-resolution MS spectrum of acetonide of **4** in positive ion mode.

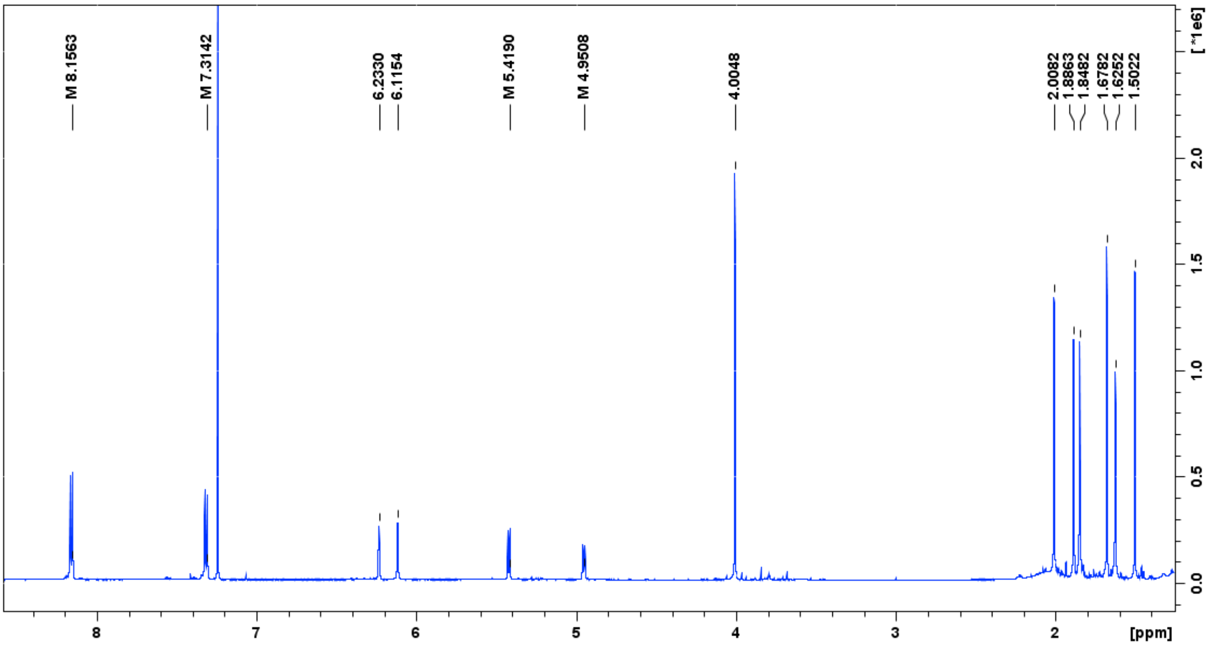

Figure S39. ^{1}H NMR spectrum of acetonide of **4** in $CDCl_3$ at 300 K.

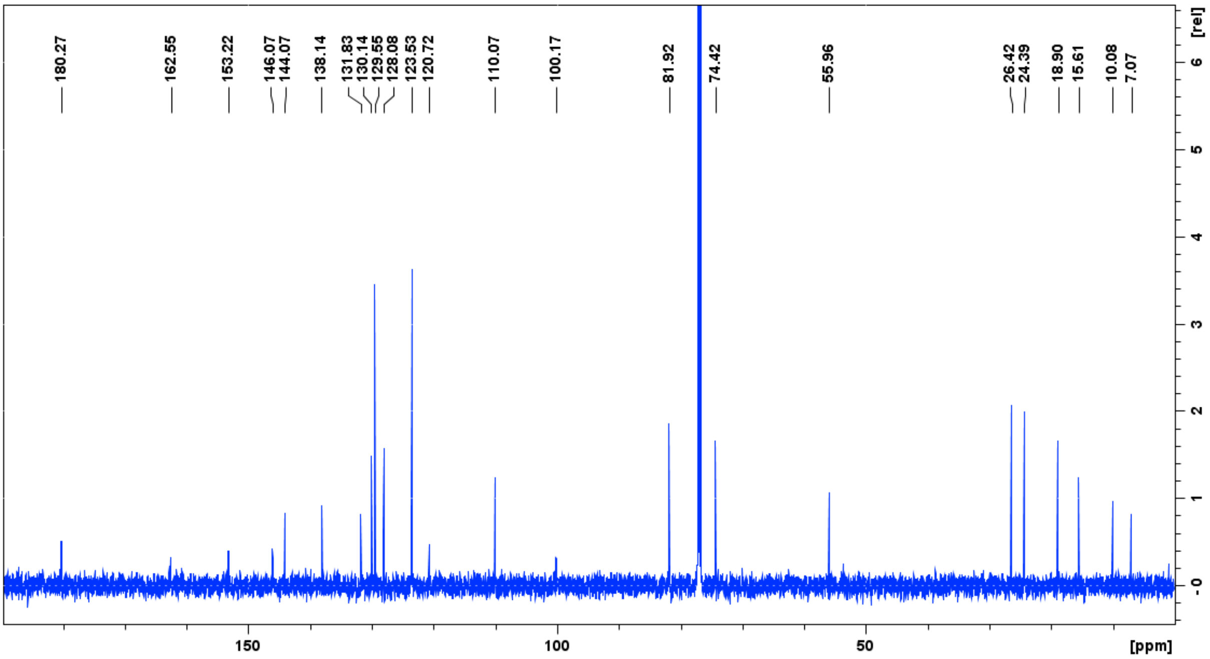

Figure S40. ^{13}C NMR spectrum of acetonide of **4** in $CDCl_3$ at 300 K.

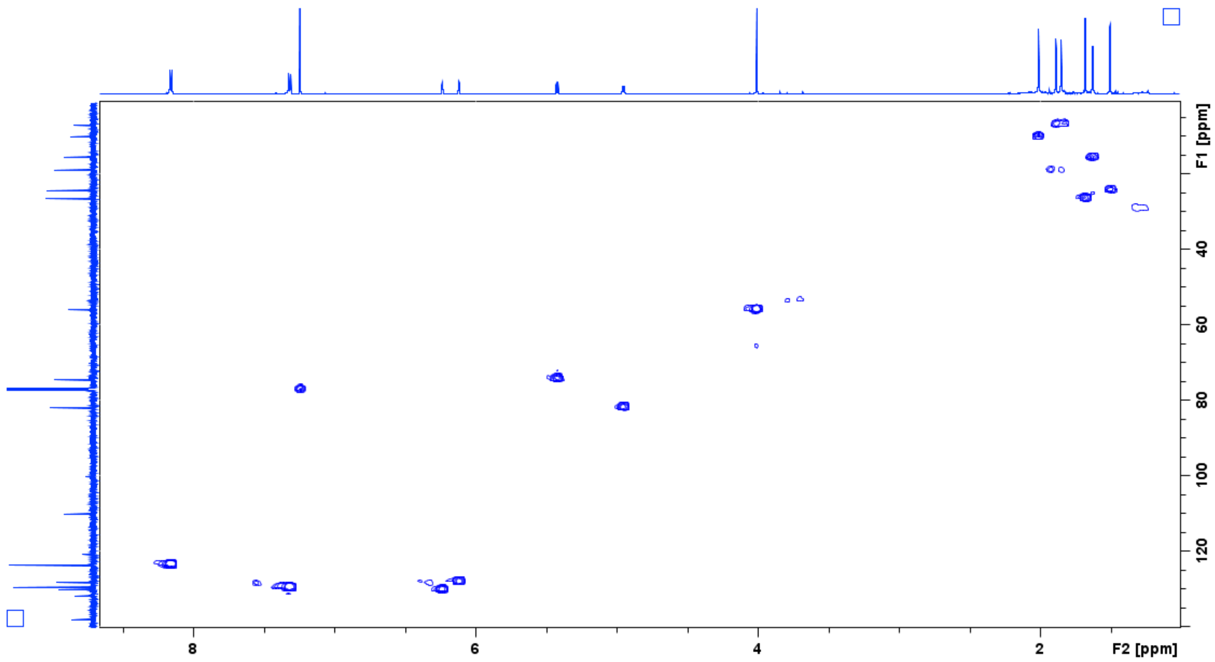

Figure S41. HSQC spectrum of acetonide of **4** in $CDCl_3$ at 300 K.

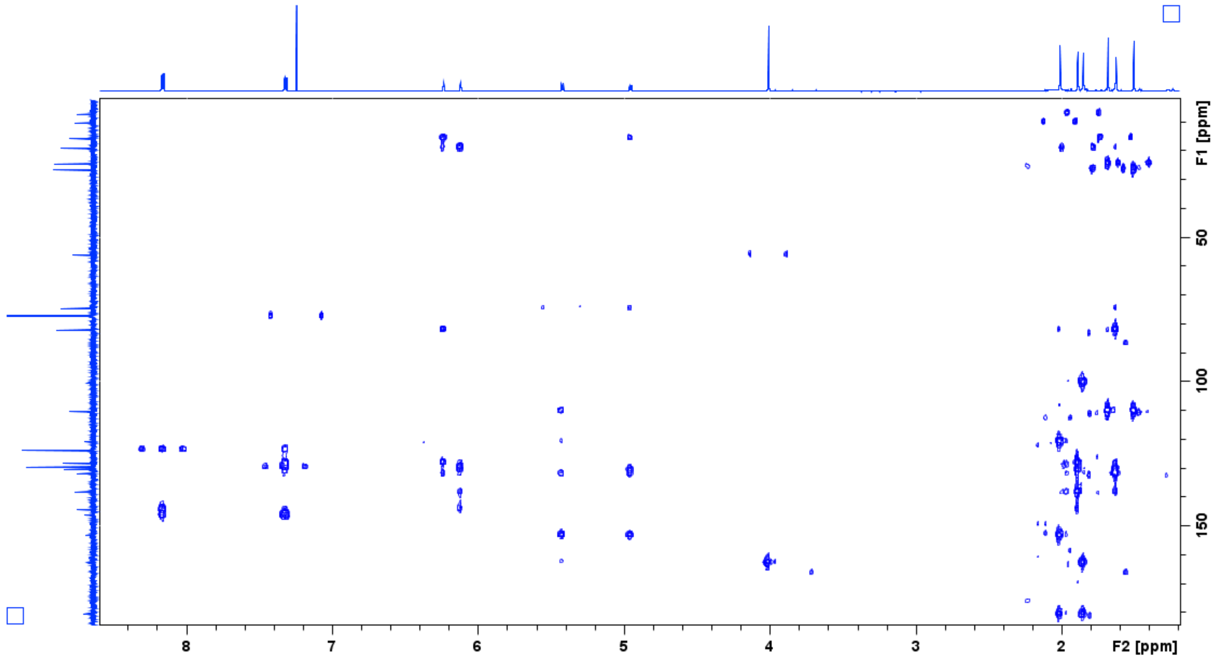

Figure S42. HMBC spectrum of acetonide of **4** in $CDCl_3$ at 300 K.

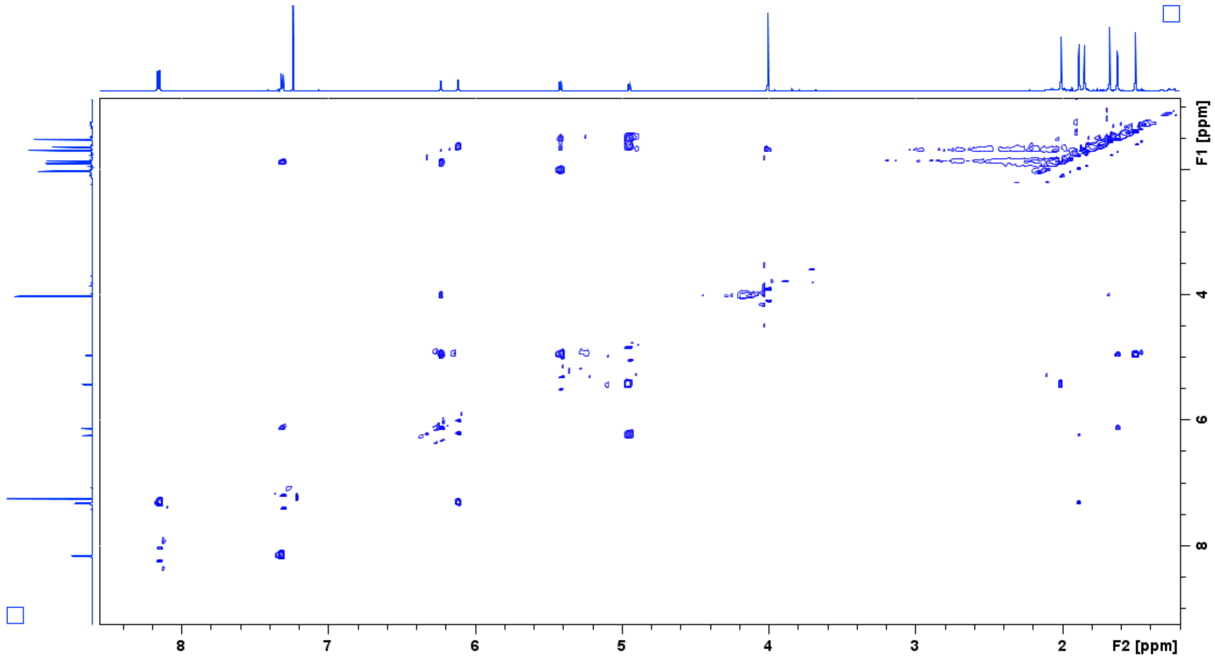

Figure S43. NOESY spectrum of acetonide of **4** in $CDCl_3$ at 300 K.

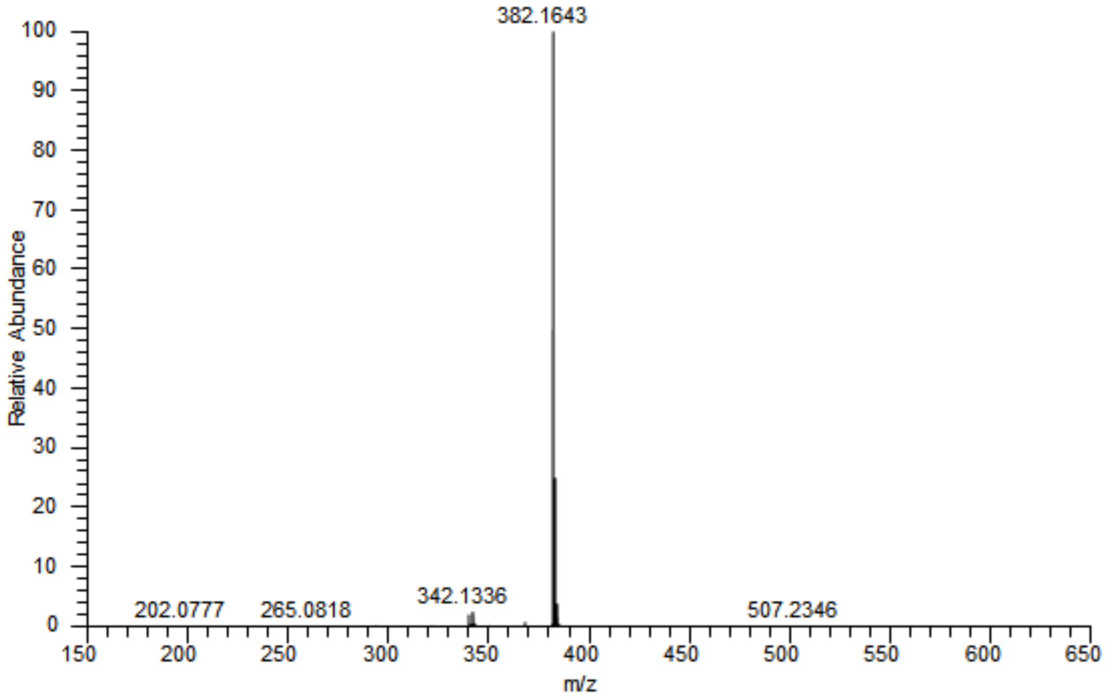

Figure S44. High-resolution MS spectrum of **6** in positive ion mode.

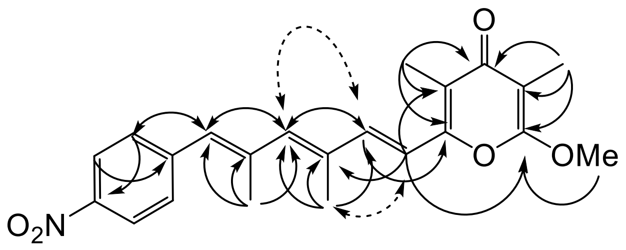

Figure S45. Selected HMBC (arrows) and ROESY (dashed arrows) correlations of **6**.

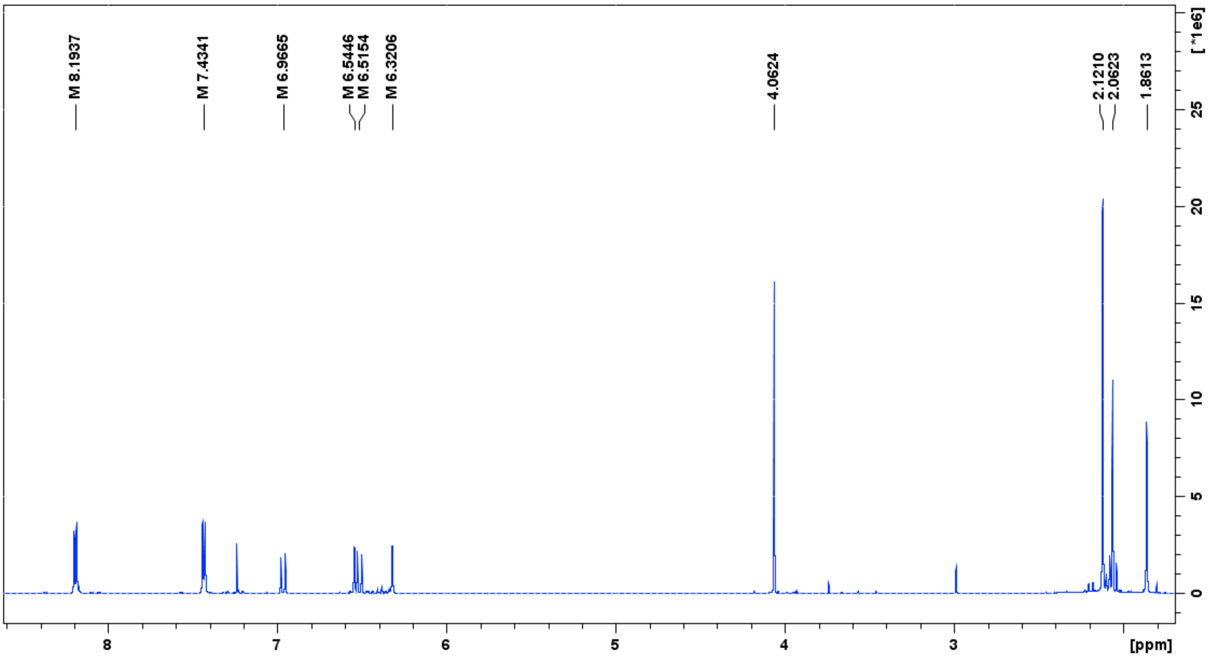

Figure S46. ^1H NMR spectrum of 7-dehydro-7-deoxyaureothin (**6**) in $CDCl_3$ at 300 K.

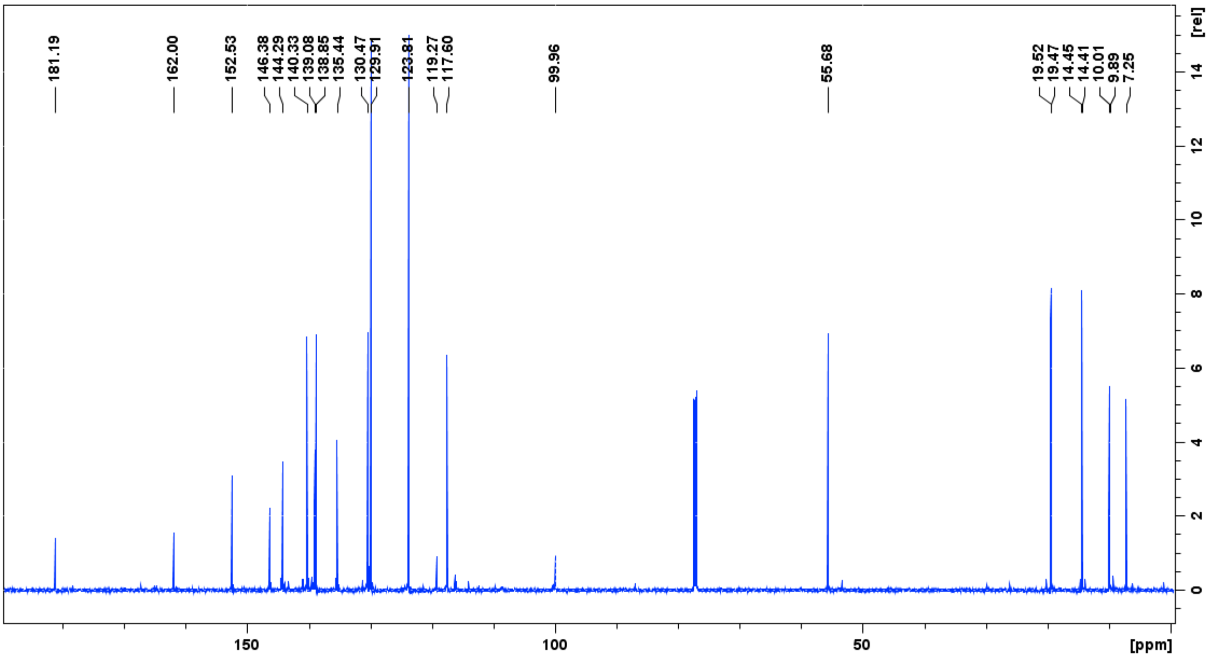

Figure S47. ^{13}C NMR spectrum of 7-dehydro-7-deoxyaureothin (**6**) in $CDCl_3$ at 300 K.

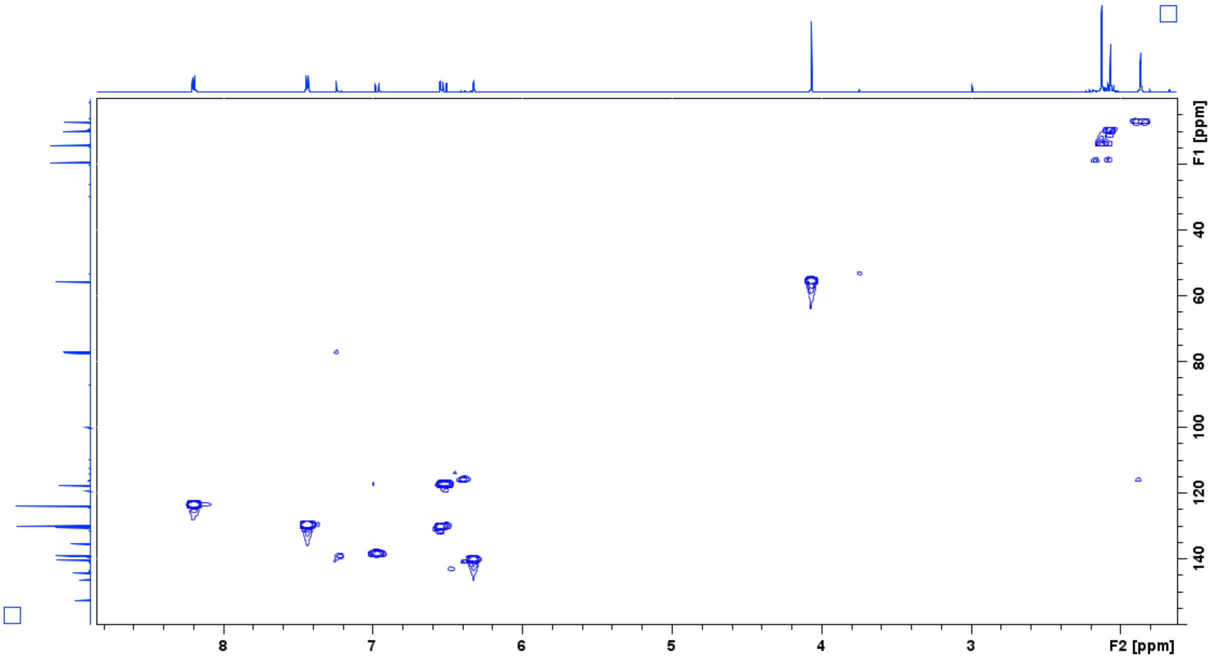

Figure S48. HSQC spectrum of 7-dehydro-7-deoxyaureothin (**6**) in $CDCl_3$ at 300 K.

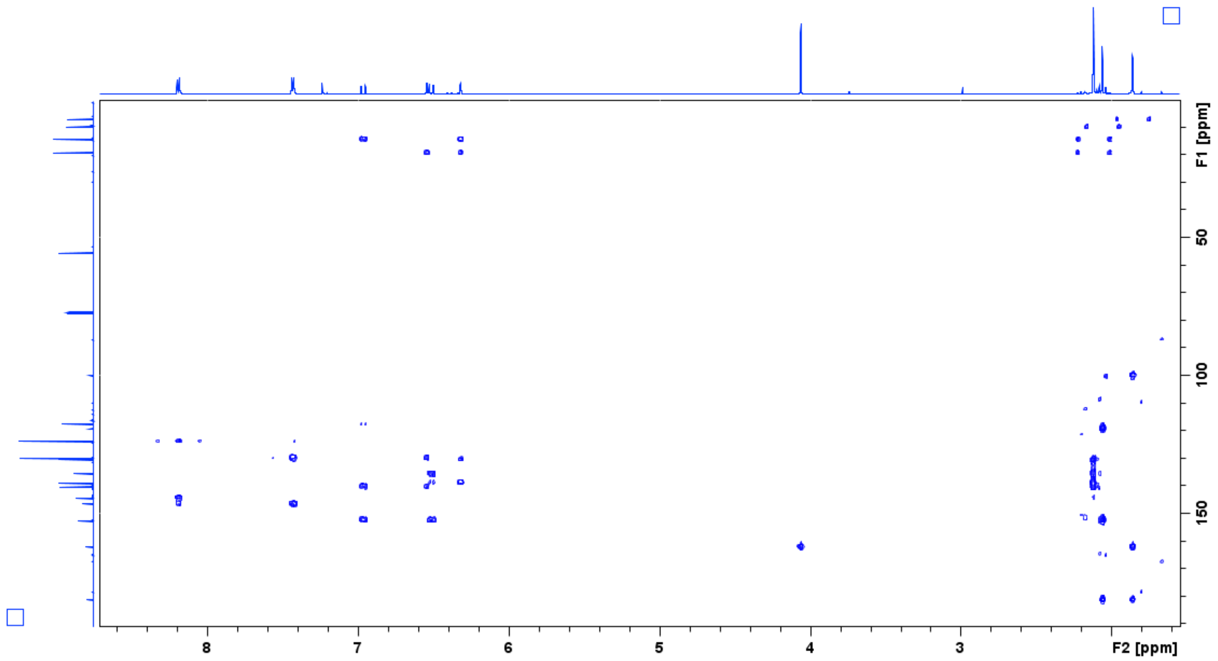

Figure S49. HMBC spectrum of 7-dehydro-7-deoxyaureothin (**6**) in $CDCl_3$ at 300 K.

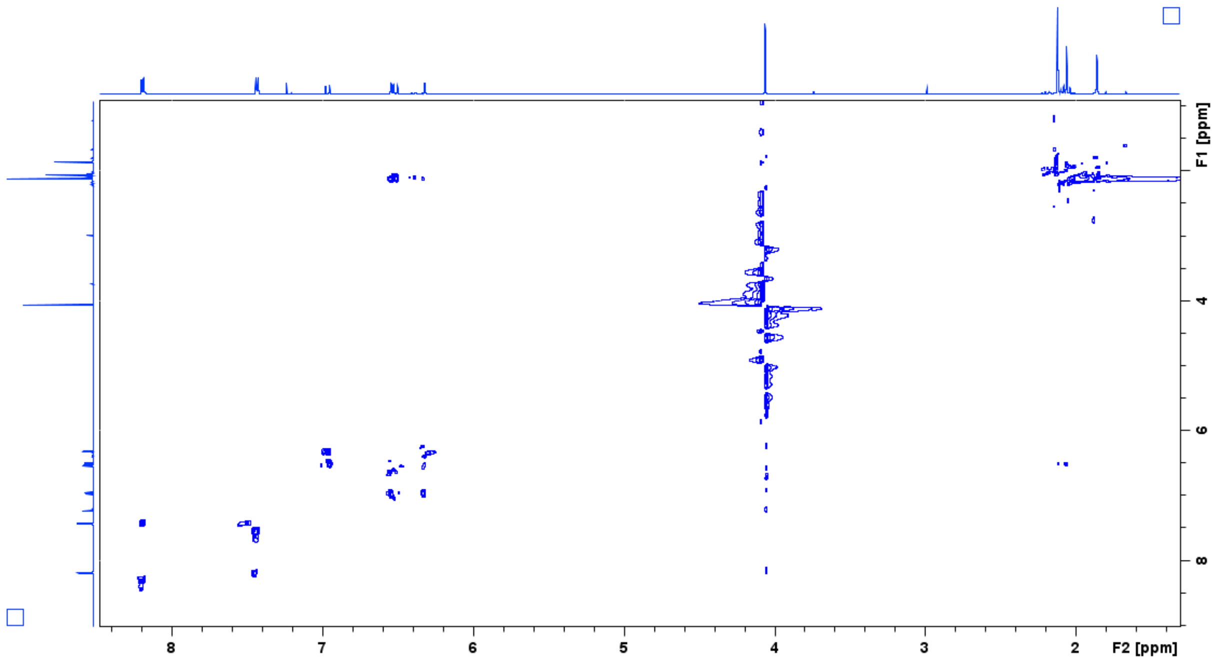

Figure S50. ROESY spectrum of 7-dehydro-7-deoxyaureothin (**6**) in $CDCl_3$ at 300 K.

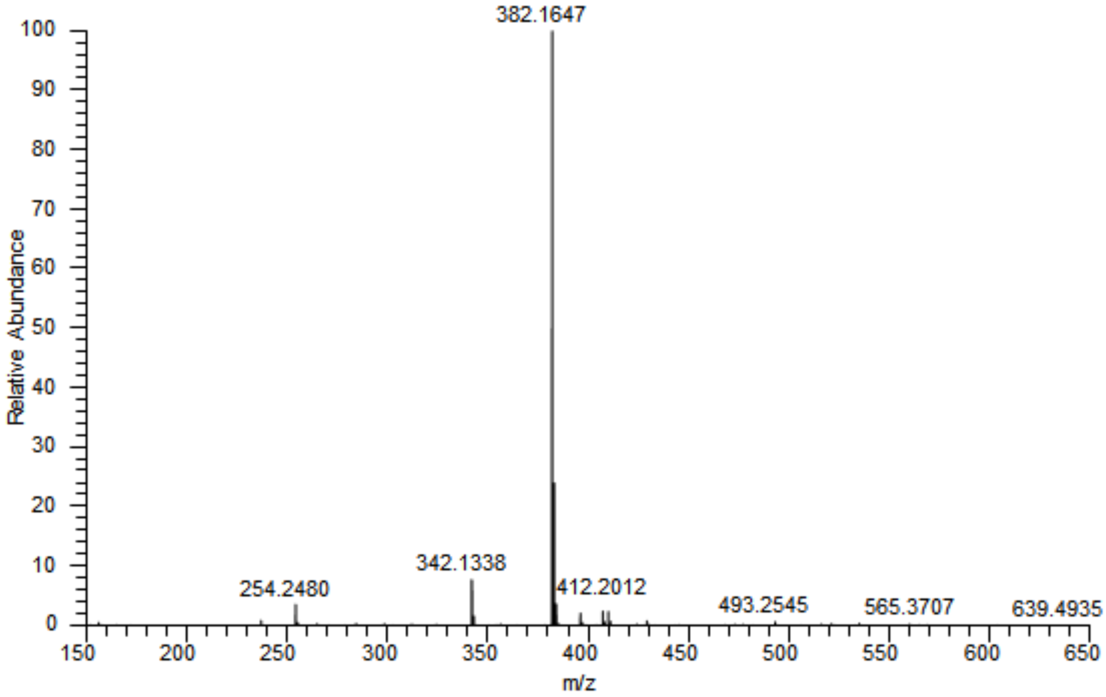

Figure S51. High-resolution MS spectrum of **7** in positive ion mode.

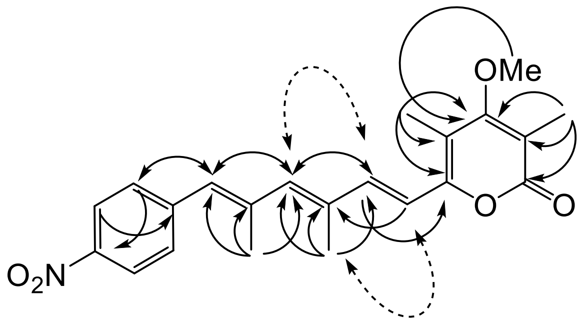

Figure S52. Selected HMBC (arrows) and ROESY (dashed arrows) correlations of **7**.

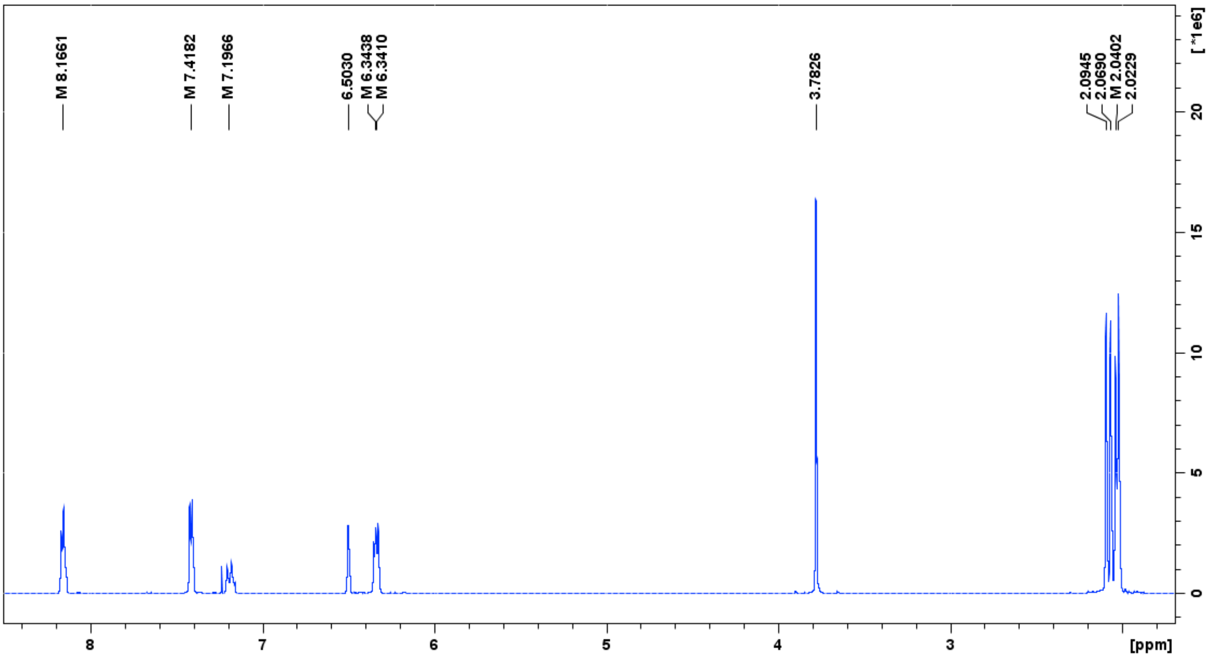

Figure S53. ^{1}H NMR spectrum of 2-pyrone-7-dehydro-7-deoxyaureothin (**7**) in $CDCl_3$ at 300 K.

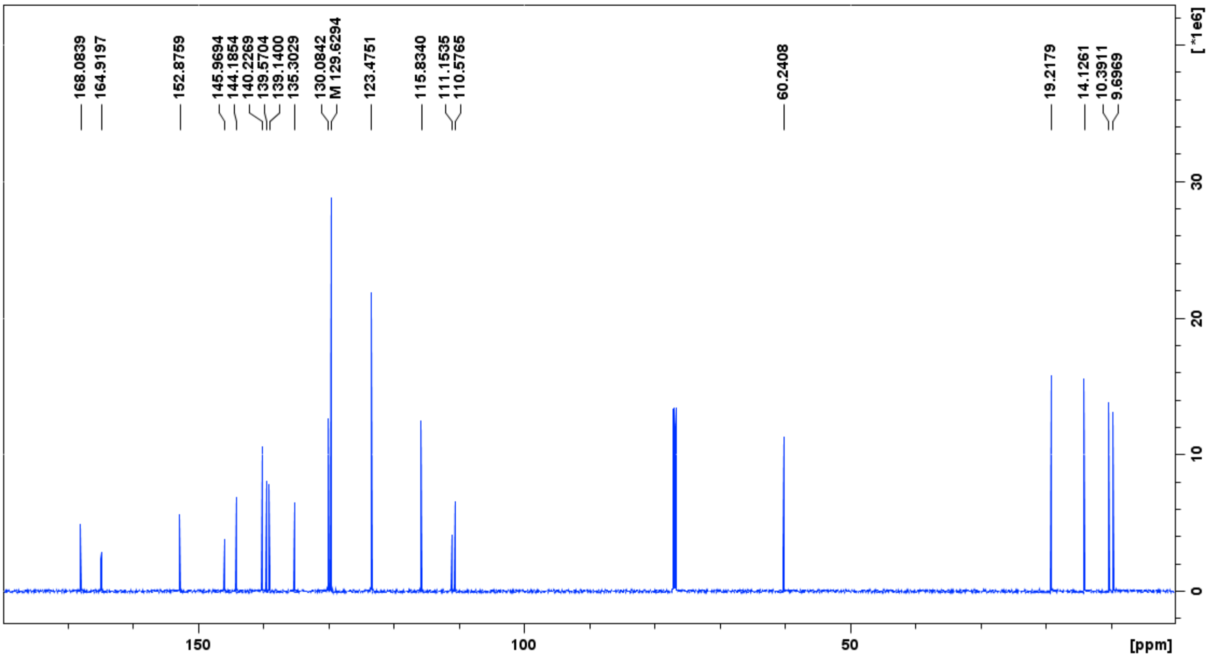

Figure S54. ^{13}C NMR spectrum of 2-pyrone-7-dehydro-7-deoxyaureothin (**7**) in $CDCl_3$ at 300 K.

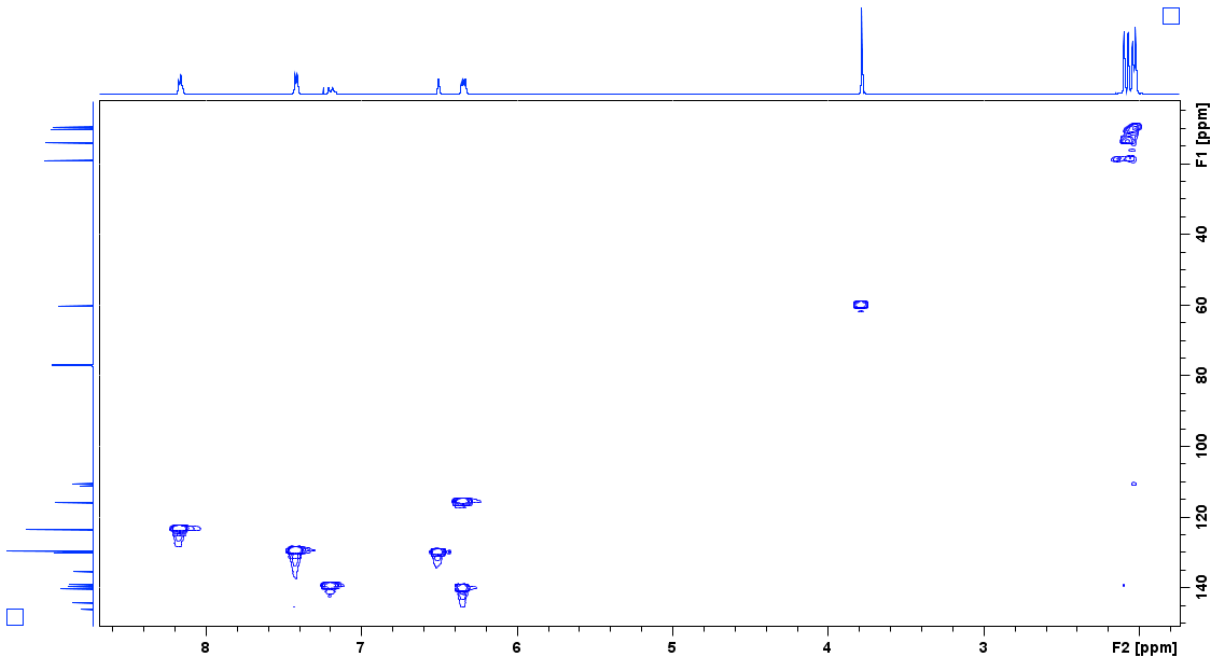

Figure S55. HSQC spectrum of 2-pyrone-7-dehydro-7-deoxyaureothin (**7**) in $CDCl_3$ at 300 K.

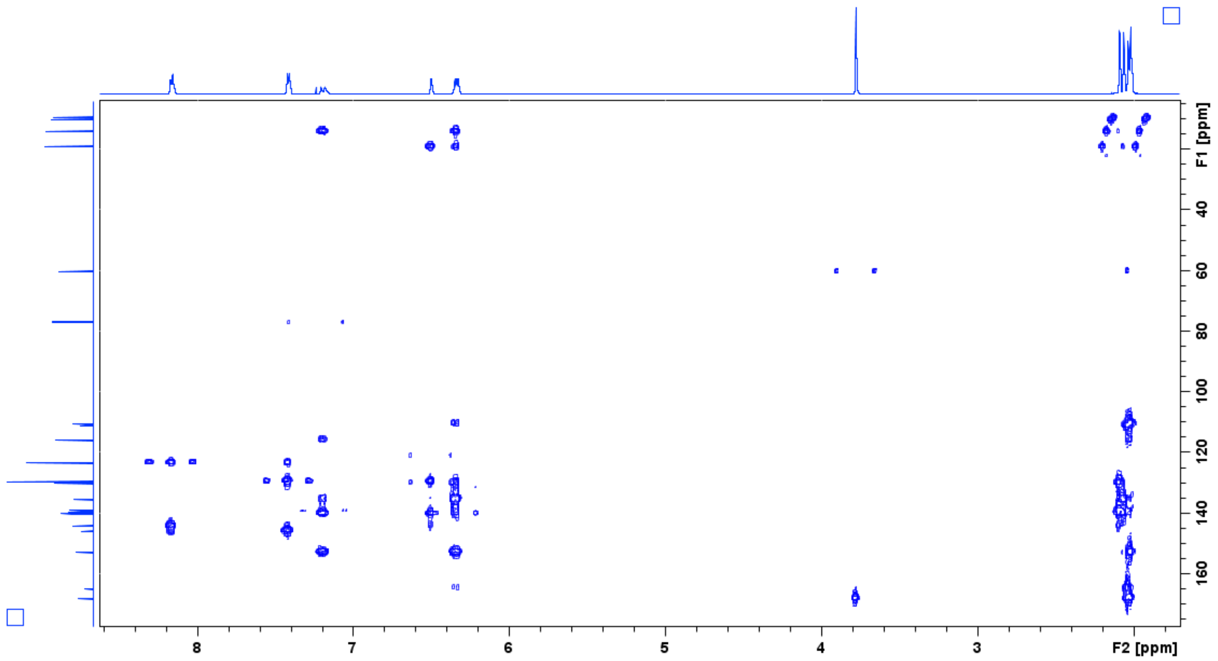

Figure S56. HMBC spectrum of 2-pyrone-7-dehydro-7-deoxyaureothin (**7**) in $CDCl_3$ at 300 K.

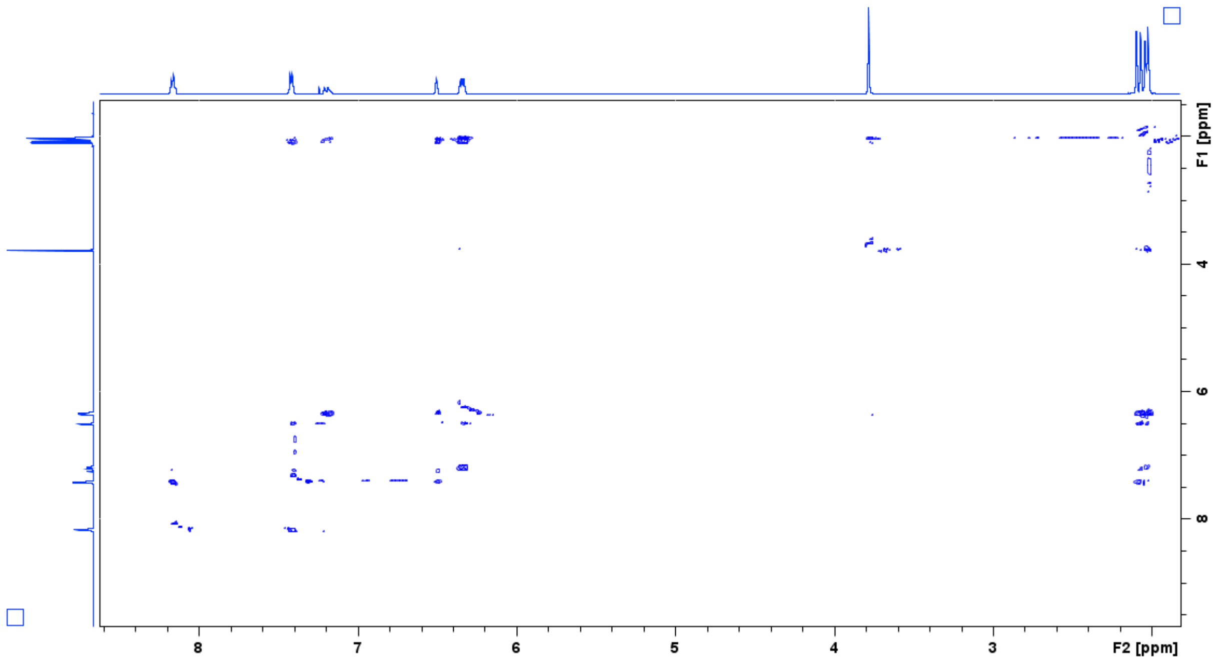

Figure S57. ROESY spectrum of 2-pyrone-7-dehydro-7-deoxyaureothin (**7**) in $CDCl_3$ at 300 K.

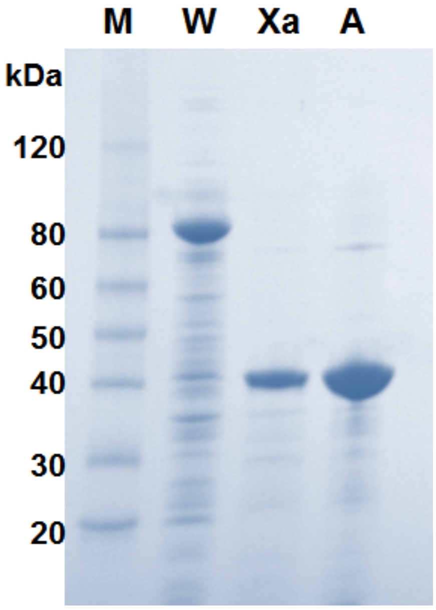

Figure S58. SDS-PAGE of AurH purification. M, molecular marker; W, whole cell (MalE-AurH fusion protein, ≈ 80 kDa); Xa, eluent (AurH protein, ≈ 40 kDa) after factor Xa digestion on amylose resin; A, eluent from *p*-aminobenzamidine resin.

Supplementary references

[1] G. Ditta, S. Stanfield, D. Corbin, D. R. Helinski, *Proc. Natl. Acad. Sci. USA* **1980**, *77*, 7347-7351.

[2] B. Gust, G. L. Challis, K. Fowler, T. Kieser, K. F. Chater, *Proc. Natl. Acad. Sci. USA* **2003**, *100*, 1541-1546.

[3] M. E. Richter, N. Traitcheva, U. Knupfer, C. Hertweck, *Angew. Chem. Int. Ed.* **2008**, *47*, 8872-8875.

[4] T. Kieser, M. J. Bibb, M. J. Buttner, K. F. Chater, D. A. Hopwood, *Practical Streptomyces Genetics*, The John Innes Foundation, Norwich, **2000**.

[5] J. Sambrook, *Molecular cloning : a laboratory manual*, Cold Spring Harbor Laboratory Press, New York, **2001**.

[6] J. He, C. Hertweck, *Chem. Biol.* **2003**, *10*, 1225-1232.

[7] Y. Sugimoto, L. Ding, K. Ishida, C. Hertweck, *Angew. Chem. Int. Ed.* **2014**, *53*, 1560-1564.

[8] W. Li, A. Cowley, M. Uludag, T. Gur, H. McWilliam, S. Squizzato, Y. M. Park, N. Buso, R. Lopez, *Nucleic Acids Res.* **2015**, *43*, W580-584.

[9] A. T. Keatinge-Clay, *Chem. Biol.* **2007**, *14*, 898-908.

[10] P. Caffrey, *ChemBioChem* **2003**, *4*, 649-662.
